# Design of Experiments Investigation of Sericin Acetylation Using a Quantitative FTIR Approach

**DOI:** 10.3390/polym18161960

**Published:** 2026-08-11

**Authors:** Rony Aad, Luca Leuzzi, Diletta Ami, Greta Bianchi, Marco Mangiagalli, Antonino Natalello, Laura Cipolla, Simone Vesentini

**Affiliations:** 1 Department of Electronics, Information, and Bioengineering, Politecnico di Milano, 20133 Milano, Italy; rony.aad@polimi.it; 2 Department of Biotechnology and Biosciences, University of Milano-Bicocca, 20126 Milano, Italy; luca.leuzzi@unimib.it (L.L.); diletta.ami@unimib.it (D.A.); greta.bianchi@unimib.it (G.B.); marco.mangiagalli@unimib.it (M.M.); antonino.natalello@unimib.it (A.N.)

**Keywords:** sericin, acetylation, Design of Experiments (DoE), protein functionalization, quantitative FTIR analysis, β-sheet structure, bio-based materials

## Abstract

Sericin, a silk-derived protein recovered as a by-product of the textile industry, is a renewable biomacromolecule with considerable potential for sustainable material development. Chemical modification represents an effective strategy for its valorization. In this study, acetylation using acetyl chloride (AcCl) was selected as a model reaction to systematically investigate the reactivity of sericin in an *N*,*N*-dimethylacetamide/lithium chloride (DMA/LiCl) solvent system and to evaluate the influence of reaction parameters on both the extent of functionalization and protein secondary structure. A Design of Experiments (DoE) strategy, comprising an initial full factorial screening followed by Box–Behnken optimization, was employed to investigate the effects of AcCl equivalents, sericin concentration, and LiCl content. A quantitative FTIR workflow based on constrained Gaussian deconvolution was developed to derive functionalization index (FI) and β-sheet index (BI) from both peak areas and peak intensities, enabling the simultaneous evaluation of chemical modification and structural organization. The exploratory screening identified AcCl as the dominant factor governing sericin functionalization. During the optimization phase, the FI models described the general response trends, whereas the BI was successfully represented by robust quadratic models (R^2^ = 0.977–0.978; adjusted R^2^ = 0.936–0.938), revealing significant linear, interaction, and quadratic effects, with LiCl concentration and sericin concentration playing key roles in governing structural organization. The analytical workflow was verified by reproducibility assessment and independent validation experiments. Overall, this study proposes a quantitative DoE–FTIR framework for systematically investigating sericin functionalization and its associated structural evolution, providing support for future studies aimed at sericin industrial valorization.

## 1. Introduction

Sericin is a silk-derived protein that accounts for approximately 20–30% of silk fiber mass and is generated in large quantities as a by-product of silk processing. Sericin is rich in hydrophilic amino acid residues, with serine being the most abundant. Consequently, it contains a high density of hydroxy groups together with amino and carboxy functionalities, making it an attractive substrate for chemical modification. Owing to its biocompatibility, biodegradability, antioxidant activity, and broad application potential in biomedical, cosmetic, food, and materials science fields, sericin has attracted considerable attention as a sustainable bio-based material [1,2,3,4,5,6].

Despite these advantages, sericin exhibits several limitations that restrict its use as a material platform. Although readily soluble in water, it displays limited solubility in most organic solvents due to extensive intermolecular hydrogen bonding and protein–protein interactions arising from its highly polar composition. In addition, sericin often exhibits high hydrophilicity, hygroscopicity, limited mechanical strength, and stability concerns [7]. Its physicochemical behavior is further influenced by molecular weight distribution and secondary structure, particularly the balance between random coil and β-sheet conformations.

To overcome these limitations, various modification strategies have been explored, including blending with other polymers, chemical modification, and functionalization [8]. Among these approaches, chemical functionalization represents a particularly attractive route for tailoring sericin properties by altering its chemical composition and intermolecular interactions. Through controlled functionalization, improvements in solubility, structural stability, and material performance may be achieved.

Considerable research has been devoted to the preparation of chemically modified sericin derivatives, and numerous studies have reported successful functionalization strategies aimed at tailoring its physicochemical and biological properties [9,10,11,12,13]. However, despite this extensive literature, systematic investigations of sericin reactivity remain limited. In particular, studies correlating reaction parameters with functionalization extent and protein structural organization are relatively scarce. Furthermore, quantitative methodologies capable of reproducibly monitoring these changes and supporting statistically guided process optimization are not commonly employed. As a result, direct comparison between studies and rational optimization of sericin functionalization remain challenging.

In this context, acetylation was selected as a model functionalization reaction. Acetylation is chemically straightforward, highly reproducible, and targets abundant nucleophilic functionalities naturally present in sericin, particularly hydroxy groups of serine and threonine residues and amino groups of lysine residues. Moreover, the introduction of relatively small acetyl groups minimizes steric effects while producing measurable changes in polarity, intermolecular interactions, solubility behavior, and protein organization. These characteristics make acetylation a suitable model system for systematically investigating sericin reactivity.

It was hypothesized that the extent of sericin acetylation and the associated changes in protein structural organization are governed by both the individual and interactive effects of the reaction parameters. Specifically, variations in acetyl chloride (AcCl) amount, sericin concentration (Ser), and lithium chloride (LiCl) content in the DMA/LiCl solvent system were expected to produce measurable changes in both functionalization and secondary structure that could be quantitatively described using a Design of Experiments (DoE) framework.

Accordingly, the objective of this study was to investigate sericin acetylation using a DoE approach combined with quantitative FTIR analysis. The effects of AcCl amount, Ser concentration, and LiCl content on both functionalization and protein structural organization were systematically evaluated through FTIR-derived response variables and statistical modeling. The proposed methodology was further used to explore the relationships between reaction conditions, functionalization extent, secondary structure evolution, and solubility behavior.

## 2. Materials and Methods

### 2.1. Materials

Fresh Bombyx mori silk cocoons, including first-grade, second-grade, and pure sericin cocoons [14], were provided by the Council for Agricultural Research and Economics (CREA, Rome, Italy). A chemically degummed sericin sample was kindly provided by Cosetex S.r.l. (Milan, Italy) and was used for the comparative characterization studies. The cocoon preparation process included classification, cutting, manual removal of pupae, and storage of the cocoon shells under controlled dry conditions at room temperature until further use to preserve material quality [14]. Acetyl chloride (AcCl, ≥99%), *N*,*N*-dimethylacetamide (DMA, ≥99%), lithium chloride (LiCl, ≥99%), and acetone (≥99.5%) were purchased from Sigma-Aldrich (St. Louis, MO, USA) and used as received.

### 2.2. General Procedure for Sericin Acetylation

Native sericin (hereafter referred to as sericin) was extracted from second-grade Bombyx mori silk cocoons using the high-temperature high-pressure (HTHP) method as previously reported by our group [14]. Briefly, cocoon shells were autoclaved in water at 121 °C for 60 min; the extract was filtered, spray-dried, and stored until use.

In a typical DoE, 0.5 g of sericin was dissolved in 50.0, 16.67, or 10.0 mL of LiCl/DMA solution containing 1–10% (*w*/*v*) LiCl and heated at 90 °C for 2 h under magnetic stirring until homogeneous solutions were obtained, corresponding to sericin concentrations of 0.01, 0.03, and 0.05 g/mL, respectively. After cooling to room temperature, AcCl was added in amounts corresponding to 5, 27.5, or 50 equivalents relative to the estimated molar equivalents of nucleophilic groups in sericin. The number of reactive groups was estimated assuming that approximately one-third of the amino acid residues bear nucleophilic functionalities (mainly serine, threonine, and lysine). Accordingly, for the fixed sericin mass of 0.50 g used in all experiments, the mass of potentially reactive groups was estimated as 0.165 g, corresponding to 1.57 mmol based on an average amino acid residue molecular weight of 105.45 g/mol. The acetyl chloride levels (5, 27.5, and 50 equivalents) were then calculated relative to this estimated amount, providing a consistent normalization framework for comparing the experimental conditions within the DoE rather than an exact quantification of the accessible reactive sites. The detailed calculation procedure is provided in Section S3.1 of the Supplementary Materials. The reaction mixture was stirred at room temperature for 24 h and subsequently precipitated in a ten-fold excess of ice-cold acetone. The precipitate was recovered by centrifugation and purified through sequential washing cycles with acetone followed by distilled water to remove soluble reaction by-products, residual solvent, salts, and unreacted reagents. No separate neutralization step was performed after the reaction. The purified material was subsequently air-dried for 24 h at room temperature (22–25 °C) under a fume hood. The final removal of residual solvent was achieved using a stream of purified dry air prior to FTIR analysis, following a standard protocol commonly employed for protein FTIR measurements and described in detail by Ami and Natalello [15]. All samples underwent the same purification, drying, handling, storage, and analytical procedures to ensure consistent post-reaction treatment throughout the experimental design.

### 2.3. Design of Experiments (DoE)

The experimental design, data processing, and statistical analyses were carried out using JMP software (version 18.2.1, JMP Statistical Discovery LLC, Cary, NC, USA). The adequacy of the generated models was assessed based on the coefficient of determination (R^2^) and the adjusted coefficient of determination (R^2^adj). Statistical significance of model coefficients was evaluated using analysis of variance (ANOVA) where an independent estimate of experimental error was available. For the optimization models, coefficients with *p*-values lower than 0.05 were considered statistically significant.

Three independent continuous variables were considered in the experimental design: (i) sericin concentration (g/mL), (ii) acetyl chloride equivalents (AcCl, equiv.), and (iii) lithium chloride (LiCl) concentration (*w*/*v* % in DMA). The investigated ranges and corresponding coded values for the DoE designs are summarized in Table 1. The design space was expanded between the screening and optimization phases, with the upper level of LiCl in the screening design corresponding to the central level in the optimization design, enabling broader exploration of the experimental domain. The AcCl equivalents were defined relative to the estimated molar equivalents of reactive functional groups in sericin. The number of reactive sites was approximated by assuming that roughly a third (33%) of amino acid residues bear nucleophilic functionalities (e.g., hydroxy groups of serine and threonine, and primary amines of lysine). Accordingly, one equivalent of AcCl corresponds to the amount required to react with one mole of nucleophilic groups. LiCl concentration was expressed as weight percentage relative to the DMA solvent, thereby defining the composition of the DMA/LiCl reaction medium investigated throughout the experimental design.

The selected factor ranges were chosen to capture relevant variations in reaction conditions. AcCl was varied from 5 to 50 equivalents, spanning conditions from moderate excess, sufficient to ensure measurable functionalization despite limited accessibility of reactive sites, to large excess capable of compensating for competing hydrolysis and approaching saturation conditions. Sericin concentration was varied from 0.01 to 0.05 g/mL to balance solubility and processability constraints while enabling evaluation of the effect of protein content on reaction efficiency. LiCl content ranged from 1 to 10 wt% relative to the solvent to investigate its role in modulating protein solvation and disrupting intermolecular interactions that influence the accessibility of reactive sites.

A two-level full factorial design (2^3^, eight experimental runs without replicates) was employed for the initial exploratory screening phase. This saturated design allows the simultaneous evaluation of three key factors using a minimal number of experiments, making it particularly suitable for identifying the most influential variables within a system. Because this unreplicated design has zero residual degrees of freedom, it does not provide an independent estimate of experimental error and, therefore, does not permit formal statistical significance testing. Accordingly, the FFD was used exclusively as an exploratory screening tool to identify the factors to be investigated in the subsequent Box–Behnken optimization.

The response of the system was evaluated using two quantitative descriptors derived from FTIR analysis: the functionalization index (FI), reflecting the extent of acetylation, and the β-sheet index (BI), describing changes in protein secondary structure. Each response was calculated using both Gaussian peak areas and corresponding peak intensities, resulting in four responses: FI (area), FI (intensity), BI (area), and BI (intensity).

Experiments were performed in randomized order as generated by JMP Pro. Regression analysis was carried out using ordinary least squares (OLS), and the system was described using a linear model including main effects and interaction terms:

$$y=\beta_{0}+\beta_{1}X_{1}+\beta_{2}X_{2}+\beta_{3}X_{3}+\beta_{12}X_{1}X_{2}+\beta_{13}X_{1}X_{3}+\beta_{23}X_{2}X_{3}+\beta_{123}X_{1}X_{2}X_{3}$$

where y represents the response variable; $\beta_{0}$ is the intercept; $\beta_{i}$ are the coefficients associated with the linear effects of factors, $X_{1}{,X}_{2},\mathrm{and}\;X_{3}$; $\beta_{ij}$ represent the two-factor interaction coefficients; and $\beta_{123}$ corresponds to the three-factor interaction coefficient.

The degrees of freedom (DoF) are defined as:

DoF = N−P−R

where N represents the number of performed experiments, P the number of model equation terms (including the intercept), and R the number of replicates.

In the present case, the design comprised eight experiments and eight model equation terms (intercept, three main effects, three two-factor interactions, and one three-factor interaction), resulting in zero degrees of freedom (8 − 8 − 0 = 0). Consequently, no independent estimate of experimental error could be obtained, which is a known limitation of saturated screening designs. Therefore, the statistical indicators reported at this stage should not be interpreted as conventional significance tests but rather as exploratory measures of relative factor importance. Accordingly, the primary objective of the screening study was to identify potentially influential factors, reveal trends, and establish a ranking of factor effects to guide the subsequent optimization phase.

**Figure 1:**
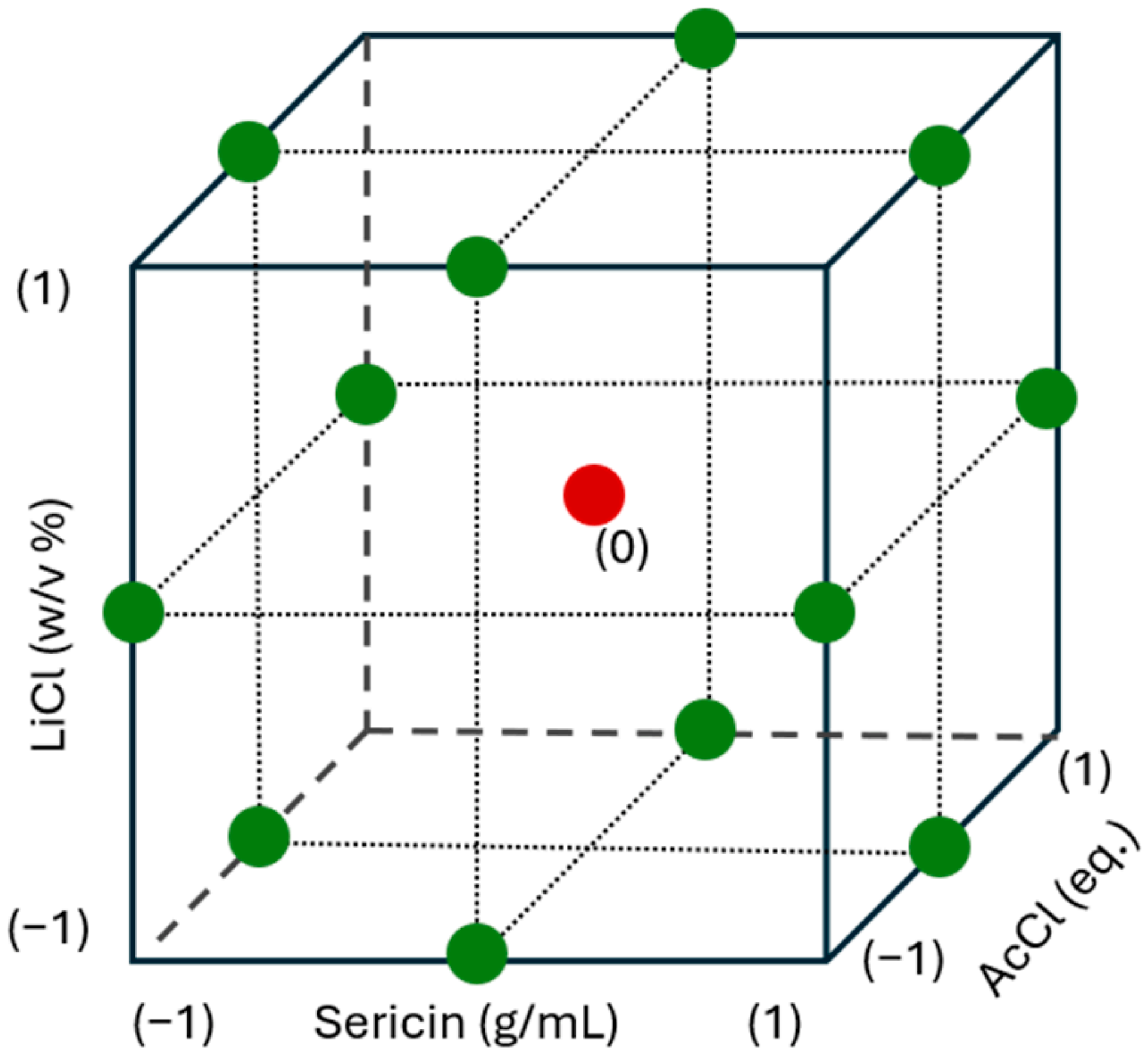
Graphical representation of the three-factor Box–Behnken design (BBD) used in this study. Experimental points (green) are located at the midpoints of the edges of the design space, while the center point (red) corresponds to the coded level (0, 0, 0) and was replicated to estimate experimental error and assess reproducibility.

The Box–Behnken design (BBD) was employed for the optimization phase and included 15 experimental runs, including three center points. This response surface methodology (RSM) enables efficient modeling of second-order (quadratic) response surfaces, including linear, two-factor interaction, and quadratic effects, while requiring fewer experiments than a three-level full factorial design. The structure of the three-factor BBD is illustrated in Figure 1. In this design, experimental points are located at the midpoints of the edges of the experimental domain, along with replicated center points, while combinations corresponding to extreme conditions (corner points) are not included. This configuration enables efficient estimation of the second-order polynomial model (linear, two-factor interaction, and quadratic terms) while avoiding experimental conditions that may result in unstable reaction conditions, excessive reagent consumption, or experimental conditions of limited practical interest for the present acetylation system.

In addition, the inclusion of replicated center points allows the estimation of pure experimental error, the assessment of experimental reproducibility, and the detection of potential non-linear behavior in the system. Regression analysis was performed using ordinary least squares (OLS), and the relationship between the response variable and the investigated factors was described using a second-order polynomial model, expressed as:

$$y=\beta_{0}+\beta_{1}X_{1}+\beta_{2}X_{2}+\beta_{3}X_{3}+\beta_{12}X_{1}X_{2}+\beta_{13}X_{1}X_{3}+\beta_{23}X_{2}X_{3}+\beta_{11}X_{1}^{2}+\beta_{22}X_{2}^{2}+\beta_{33}X_{2}^{3}+\varepsilon$$

where y represents the response variable; $\beta_{0}$ is the intercept; $\beta_{i}$ correspond to the linear effects of the factors, $X_{1}{,X}_{2},$ $\beta_{ij}$ describe the interaction effects; $\beta_{ii}$ represent the quadratic terms accounting for curvature in the response surface; and ε denotes the residual error.

### 2.4. Characterization

Sericin was characterized by ATR-FTIR spectroscopy, size-exclusion chromatography (SEC), SDS-PAGE, and circular dichroism (CD) prior to acetylation. Acetylated sericin samples were analyzed by ATR-FTIR spectroscopy.

The molecular weight distribution of sericin was analyzed by size-exclusion chromatography (SEC). Sericin powders were dissolved in phosphate-buffered saline (PBS; NaCl 150 mM, NaP 25 mM, pH 7.0) at a nominal concentration of 2 mg/mL. Samples were centrifuged to remove aggregated protein and obtain clarified solutions before analysis. Chromatographic separation was performed as described in [14]. Relevant 1 mL fractions were collected, lyophilized and stored at −20 °C.

Lyophilized sericin fractions obtained from SEC were resuspended in 180 µL of ultrapure water and clarified by centrifugation. and analyzed by sodium dodecyl sulfate–polyacrylamide gel electrophoresis (SDS-PAGE) using 14% resolving gels under denaturing conditions. Electrophoresis was performed according to standard protocols. Molecular weight estimation was carried out using the StoS Protein Marker (GeneSpin, Milano, Italy) as a reference.

Far-UV circular dichroism (CD) spectra of sericin were recorded in the 190–260 nm range using a Jasco J-815 spectropolarimeter (Jasco Corp., Tokyo, Japan). Measurements were performed in a 0.1 cm path-length quartz cuvette at room temperature. Spectra were averaged over three scans, followed by buffer subtraction and smoothing [16].

ATR-FTIR spectroscopy was used to investigate the structural features of sericin and acetylated sericin, with particular emphasis on protein secondary structure and the identification of carbonyl functionalities associated with acetylation. ATR-FTIR spectra were recorded and analyzed as previously described [15,17]. Compared with CD spectroscopy, which may be affected by artifacts in the presence of non-homogeneous suspensions [18], mid-infrared ATR-FTIR spectroscopy is less sensitive to light-scattering effects and was therefore used to compare all samples of interest under exactly the same measurement conditions [15].

In particular, the Varian 670-IR spectrometer (Varian Australia Pty Ltd., Mulgrave, VIC, Australia), equipped with a nitrogen-cooled mercury cadmium telluride (MCT) detector, was employed. Measurements were performed using a single-reflection diamond ATR accessory (Quest, Specac Ltd., Pleasantville, NY, USA) with a pressure clamp to ensure consistent contact between the sample and the crystal surface. Spectra were collected in the range of 4000–700 cm^−1^ at a spectral resolution of 2 cm^−1^ with 1024 co-added scans using triangular apodization. Background spectra were acquired prior to each measurement under identical conditions. To analyze the Amide I band spectral region and resolve overlapping components, second-derivative spectra were calculated after Savitzky–Golay smoothing. FTIR data acquisition and processing were carried out using Resolutions Pro software (Version 5.4.1.3412, Varian Australia Pty Ltd., Mulgrave, VIC, Australia).

Sericin solubility was evaluated through a two-stage approach consisting of preliminary solvent screening followed by concentration-dependent assessment in selected solvents. Initial screening was performed at 1 mg/mL in water, dimethyl sulfoxide (DMSO), *N*,*N*-dimethylformamide (DMF), 1,4-dioxane, tert-butyl acetate (t-BuOAc), *N*,*N*-dimethylacetamide (DMA), triethylamine (TEA), tetrahydrofuran (THF), acetonitrile (MeCN), and a 1:1 (*v*/*v*) THF/MeCN mixture. Samples were vortexed at room temperature and qualitatively classified according to solution appearance. Solvents affording apparent solubility were further evaluated at higher solute concentrations (100–10 mg/mL). Sericin (100 mg) was dispersed in 1 mL of solvent (100 mg/mL) and incubated at 50 °C under stirring (120 rpm) for 30 min using a SKI 4 shaking incubator (ARGO LAB, Capri, Italy). If complete dissolution was not achieved, the solvent volume was increased. At each concentration, samples were equilibrated prior to visual assessment. The effect of LiCl on sericin solubility was subsequently investigated in DMA and DMSO systems by varying LiCl content and sericin concentration in order to identify conditions capable of providing homogeneous solutions suitable for acetylation reactions. The solubility of acetylated sericin was evaluated using the same qualitative screening approach at 1 mg/mL in selected solvents (water, DMSO, DMA, and DMA/LiCl systems). No concentration-dependent study was performed for the modified samples.

### 2.5. FTIR Data Processing and Quantitative Analysis

FTIR spectra were acquired as described in Section 2.4. Subsequent spectral preprocessing and quantitative analysis were performed using Orange Data Mining software (version 3.40.0, University of Ljubljana, Ljubljana, Slovenia) [19].

To determine the positions of overlapping spectral components, second-derivative analysis was applied to the FTIR spectra. Before derivative calculation, all spectra were smoothed using a Savitzky–Golay filter applied uniformly across the dataset. The analysis focused on the 1780–1580 cm^−1^ region, encompassing the Amide I band and carbonyl stretching vibrations associated with acetylated groups. Peak positions identified from the second-derivative spectra were used to define the number and approximate centers of the Gaussian components for subsequent deconvolution of the corresponding absorption spectra.

Following peak identification, the original absorption spectra were analyzed within the same spectral window. Baseline correction was applied prior to fitting to ensure consistent spectral treatment. Because the Amide I region consists of several strongly overlapping absorption bands arising from different protein secondary structures, direct unconstrained Gaussian fitting may converge to multiple mathematically acceptable solutions that differ in the estimated contribution of individual components. Therefore, second-derivative spectroscopy was used to identify the initial peak positions, and constrained fitting parameters were employed during the nonlinear optimization. Peak centers identified from the second-derivative spectra were used as the initial positions for the Gaussian components and were allowed to vary within predefined peak-specific intervals (±1 to ±5 cm^−1^) using the delta parameter available in the fitting software. Narrow constraints (±1 cm^−1^) were applied to well-resolved bands, whereas broader intervals (up to ±5 cm^−1^) were used for overlapping bands when required to obtain convergent, physically meaningful fits while ensuring that the bands identified by second-derivative analysis remained clearly distinguishable as separate components. Representative Gaussian deconvolutions for samples exhibiting low, intermediate, and high FI values are provided in the Supplementary Materials (Figure S4).

This approach reduces the number of free fitting variables, improves the robustness and reproducibility of the deconvolution procedure, and is consistent with established protocols for quantitative FTIR analysis of protein secondary structure [19,20,21,22].

Gaussian peak fitting was performed using nonlinear least-squares optimization. Peak centers were constrained according to the second-derivative results, while peak widths were restricted to prevent unrealistic broadening or narrowing of spectral components. The integrated areas of the fitted peaks were extracted from the Gaussian components to quantify the individual vibrational contributions. The same preprocessing workflow, spectral range, fitting strategy, and band-assignment criteria were applied to all spectra to ensure consistency and minimize operator-dependent variability. The quality of the deconvolution was evaluated using the reduced χ^2^ value obtained from the Gaussian fitting procedure, with the corresponding values reported in the Supplementary Materials (Tables S1 and S2).

The Amide I band components were assigned to the corresponding protein secondary structure elements according to the broadly accepted and well-established consensus in the scientific literature [15,23].

Two independent quantitative descriptors were derived from the deconvoluted FTIR spectra to evaluate both the extent of chemical functionalization and the structural evolution of sericin. These metrics were primarily based on Gaussian peak areas, with complementary evaluation using peak intensities (amplitudes) calculated from the fitted parameters.

The first descriptor, termed the functionalization index (FI), was defined as the ratio between the ester carbonyl contribution and the total Amide I region:

$$FI_{A}=\frac{A_{Carbonyl}}{A_{Amide\;I}}$$

where $A_{Carbonyl}$ represents the Gaussian peak area assigned to acetyl carbonyl vibrations (~1745 cm^−1^) and $A_{Amide\;I}$ represents the total area of Gaussian components assigned to the Amide I region (1700–1600 cm^−1^).

Higher FI values, therefore, indicate a greater extent of acetylation. In addition to area-based evaluation, an analogous ratio was calculated using peak intensities (amplitudes):

$$FI_{I}=\frac{I_{Carbonyl}}{I_{Amide\;I}}$$

where I denotes the peak amplitudes, which were then derived assuming Gaussian peak shapes according to:

$$I=\frac{A}{\sigma \sqrt{2\pi }}$$

where I is the peak amplitude, A is the peak area, and σ is the Gaussian width parameter.

The second descriptor, termed the β-Sheet index (BI), was defined as:

$$BI_{A}=\frac{A_{\beta -sheet}}{A_{Amide\;I}}$$

where $A_{Amide\;I}$ represents the total area of Gaussian components assigned to the Amide I region (1700–1600 cm^−1^) and $A_{\beta -sheet}$ represents the sum of the two areas of peaks assigned to β-sheet structures within the Amide I region (1640–1620 cm^−1^ and ~1700 cm^−1^).

Higher BI values indicate a greater relative contribution of β-sheet structures within the protein. Similarly, a corresponding intensity-based ratio was considered:

$$BI_{I}=\frac{I_{\beta -sheet}}{I_{Amide\;I}}$$

where $I_{\beta -sheet}$ and $I_{Amide\;I}$ represent the corresponding peak amplitudes.

The selected β-sheet bands correspond to spectral components commonly associated with β-sheet structures in protein FTIR analysis and have been widely reported in the literature [15,23,24,25].

The use of both low-wavenumber (~1640–1620 cm^−1^) and high-wavenumber (~1700–1690 cm^−1^) β-sheet components allows for a more representative estimation of the relative β-sheet contribution within the Amide I region.

Among the most abundant amino acids in the composition of sericin [7], aspartic acid may contribute to the band around 1700–1690 cm^−1^ through the C=O stretching vibration of its protonated carboxylic group. However, this contribution is generally observed at higher wavenumbers or near the upper boundary of the Amide I region [26]. Therefore, although a minor side-chain contribution cannot be completely excluded, the amino-acid composition of sericin does not support a predominantly side-chain origin for the resolved component near 1700 cm^−1^, which can mostly be assigned to β-sheet structures. The use of both area- and intensity-based metrics provides complementary information, as peak areas reflect overall structural contributions, while amplitudes capture local spectral variations.

## 3. Results and Discussions

### 3.1. Preliminary Physicochemical Characterization of Sericin Samples

**Figure 2:**
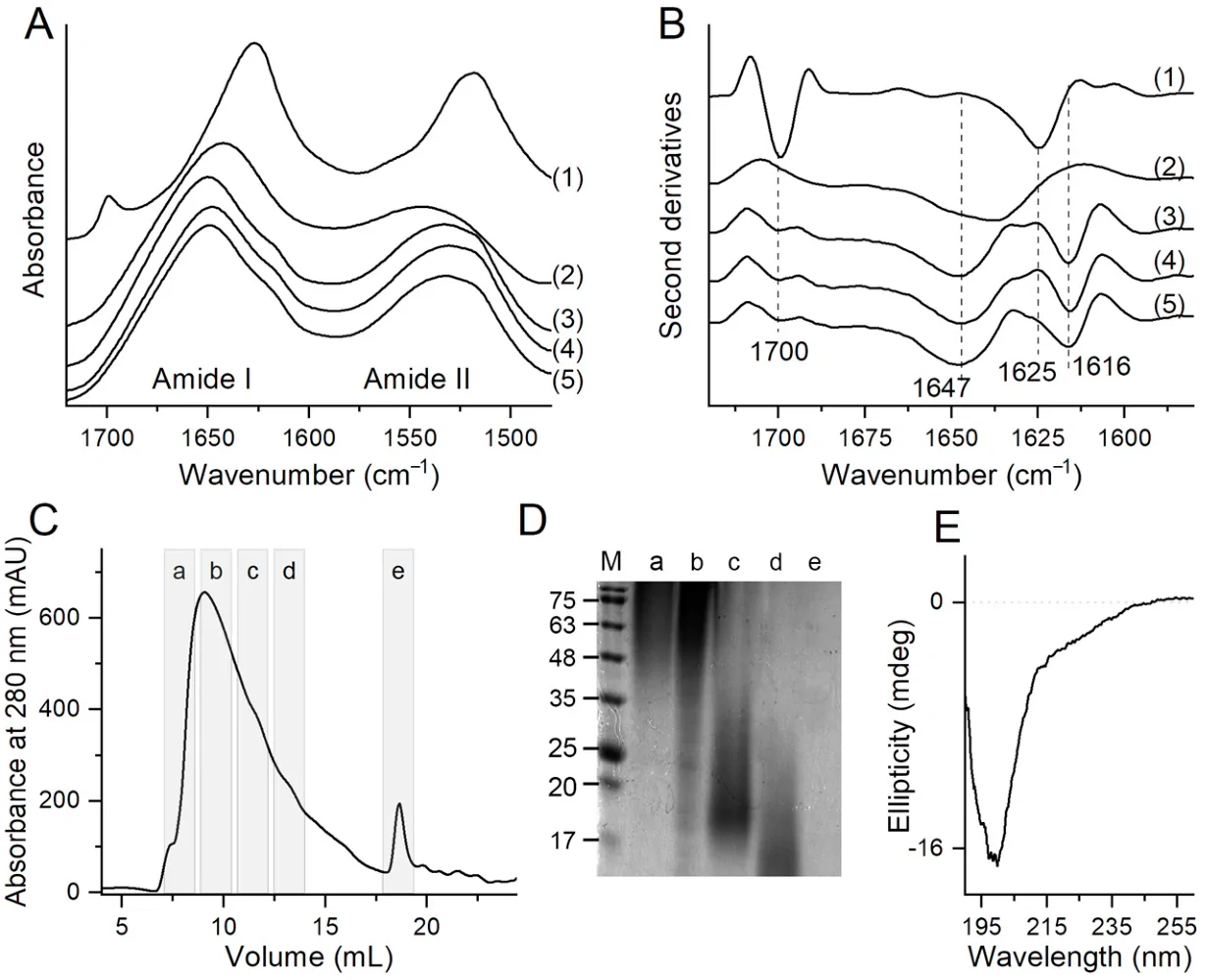
(**A**) ATR-FTIR absorption spectra of fibroin (1), chemically degummed sericin (2), first-grade cocoon sericin (3), second-grade cocoon sericin (sericin) (4), and pure sericin cocoon (5). (**B**) Second derivative of the ATR-FTIR absorption spectra reported in panel (**A**). The main peaks are indicated by the dashed vertical lines. (**C**) SEC chromatogram of sericin analyzed using a Superdex 10/200 column and PBS as the mobile phase. The collected fractions are indicated with the letters a–e. (**D**) SDS-PAGE of the collected SEC fractions; M: molecular weight marker. (**E**) Far-UV CD spectrum of sericin.

To select the most suitable sericin for acetylation studies, four samples of sericin from different sources were characterized prior to functionalization, including chemically degummed sericin (2), first-grade cocoon sericin (3), second-grade cocoon sericin (4), and pure sericin cocoon material (5). The objective was to compare their structural features and assess potential fibroin contamination by analyzing their FTIR spectra, collected in attenuated total reflection (ATR) mode, against pure fibroin as a reference (1). Fibroin and sericin display clearly distinct ATR-FTIR spectra (Figure 2A,B) owing to their structural differences. Fibroin displayed two well-resolved bands at approximately 1700 and 1625 cm^−1^, characteristic of highly ordered β-sheet structures [27]. These bands were therefore considered fibroin markers. In contrast, sericin samples exhibited a dominant broad Amide I band centered around ~1650–1645 cm^−1^, indicative of predominant random coil conformations with minor β-sheet contributions near ~1625–1616 cm^−1^ [14,23].

All the sericin samples (2–5) lacked the strong fibroin marker bands, particularly that at 1700 cm^−1^, indicating minimal fibroin contamination. In addition, samples 3–5 displayed the characteristic sericin profile, including minor β-sheet contributions, that were absent in the chemically degummed sample (2). In addition to the proteinaceous components, HTHP extraction is known to co-extract minor non-proteinaceous constituents naturally present in silk cocoons, including waxes, lipids, pigments, and inorganic salts. Although these minor constituents were not individually quantified in the present study, their possible presence in the starting sericin should be acknowledged, as they may contribute to secondary or competing reactions during subsequent acetylation. Nevertheless, the sericin was intentionally investigated in its HTHP-extracted form, without extensive post-extraction purification, to evaluate the functionalization process under conditions representative of the material as it is commonly obtained following industrial extraction.

While all samples exhibited minimal fibroin contamination, the chemically degummed sericin displayed greater structural modifications resulting from the degumming process. In contrast, sericin extracted from second-grade cocoons preserved the characteristic structural features of the sericin structural profile while remaining representative of an industrial feedstock. Moreover, second-grade cocoons are a lower-value by-product than first-grade cocoons, making their valorization both economically and environmentally attractive. Therefore, this material was selected for the subsequent characterization and acetylation studies, as it provides a suitable balance between structural preservation, industrial relevance, and valorization potential.

On this selected sample (4), the molecular weight distribution was further analyzed by SEC (Figure 2C), which revealed a broad and heterogeneous profile consistent with the polydisperse nature of sericin extracted via HTHP degumming. SDS-PAGE analysis (Figure 2D) confirmed this heterogeneity, presenting a diffuse smear across a wide molecular weight range. In addition, the SDS-PAGE analysis suggested that the peak eluted at 18 mL was a non-protein contaminant derived from the sericin preparation (fraction e in Figure 2C,D).

Finally, the far-UV CD spectra of sericin (Figure 2E) exhibited a pronounced minimum at nearly 200 nm, consistent with a predominantly disordered (random coil) conformation [28]. Minor spectral features suggested limited contributions from ordered structures, in agreement with the FTIR data. Based on its structural profile, second-grade cocoon sericin (4) was selected as the starting material for the acetylation study and is hereafter referred to as sericin. 

Prior to implementing the DoE study, a preliminary screening of sericin solubility was performed. The objective of this analysis was to define the phase behavior boundaries of the reaction medium, thereby providing a rationale for establishing the operational limits of the subsequent systematic study while minimizing potential mass transfer limitations. Preliminary screening included solvents with different polarities. The choice of solvent is known to strongly influence the dissolution of biopolymers [29,30]. Qualitative and concentration-dependent tests (Figures S1 and S2) showed that DMSO exhibited the highest dissolution capacity, while DMA and *tert*-butyl acetate provided only partial dissolution. Water was included as a reference despite its incompatibility with acylation reactions.

To further improve solubility, LiCl was introduced into the solvent system. The addition of LiCl markedly enhanced solution homogeneity in both DMA and DMSO, allowing complete dissolution of sericin at 90 °C. Although DMSO showed the highest solubilizing ability, preliminary experiments suggested possible solvent participation in side reactions [31]. Therefore, DMA/LiCl was selected as the reaction medium because it combined effective sericin dissolution with compatibility with AcCl. Following acetylation, the modified sericin exhibited reduced aqueous solubility and dissolved completely in DMSO, whereas DMA, DMF, and water produced persistent suspensions. This behavior is consistent with the reduced polarity of the protein imparted by acetylation.

### 3.2. Sericin Acetylation as a Model Reaction System

Acetylation via AcCl was selected as a model reaction owing to its well-established chemistry, cost-effective reagents, and established relevance in protein modification. The introduction of acetyl groups imparts minimal steric hindrance while effectively reducing the polarity and reactivity of protic functional groups, thereby enabling controlled modulation of protein structure. Although acetylation is a well-established reaction, its application to naturally occurring, multi-and poly-functional, and structurally heterogeneous macromolecules such as sericin results in a complex reaction system, in which multiple reactive sites, conformational variability, and competing side reactions may influence the overall outcome. Consequently, a DoE approach was employed to systematically investigate the response of the sericin acetylation system to controlled variations in the selected reaction parameters.

**Scheme 1:**
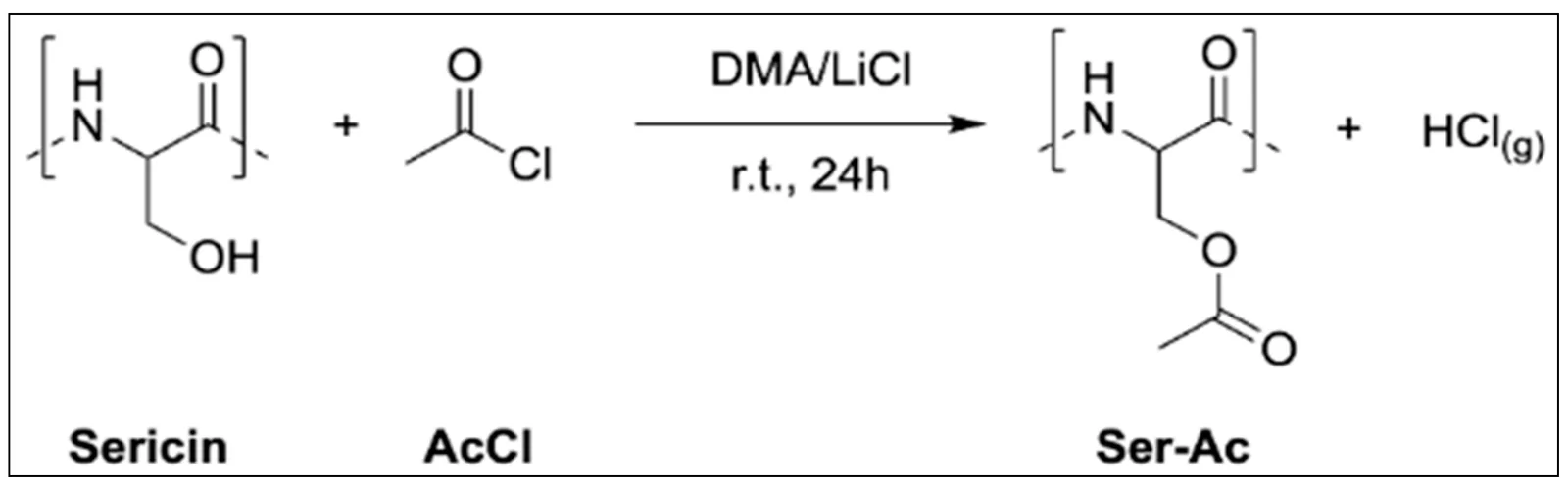
Schematic representation of sericin acetylation with AcCl in DMA/LiCl at room temperature (24 h), yielding acetylated sericin (Ser-Ac) with HCl evolution.

Sericin acetylation was carried out using AcCl in a DMA/LiCl reaction medium (Scheme 1). AcCl reacts primarily with the hydroxy groups of serine and threonine residues to form ester functionalities, although other nucleophilic side chains, including the ε-amino group of lysine and, to a lesser extent, the phenolic group of tyrosine, may also undergo acylation under the investigated conditions.

LiCl concentration was selected as an experimental factor because it determines the composition of the DMA/LiCl reaction medium and influences the physicochemical environment in which acetylation occurs [32]. Although expressing LiCl relative to protein concentration represents an alternative experimental approach, the heterogeneous and polydisperse nature of sericin makes it impractical to define a meaningful molar protein-to-salt ratio. Therefore, expressing LiCl relative to the solvent was considered a practical and reproducible approach for systematically evaluating the influence of the reaction medium within the DoE study. The acetylation system was investigated through a two-stage DoE strategy consisting of an initial exploratory screening using an FFD, followed by optimization using a BBD.

### 3.3. FTIR Characterization of Acetylated Sericin

**Figure 3:**
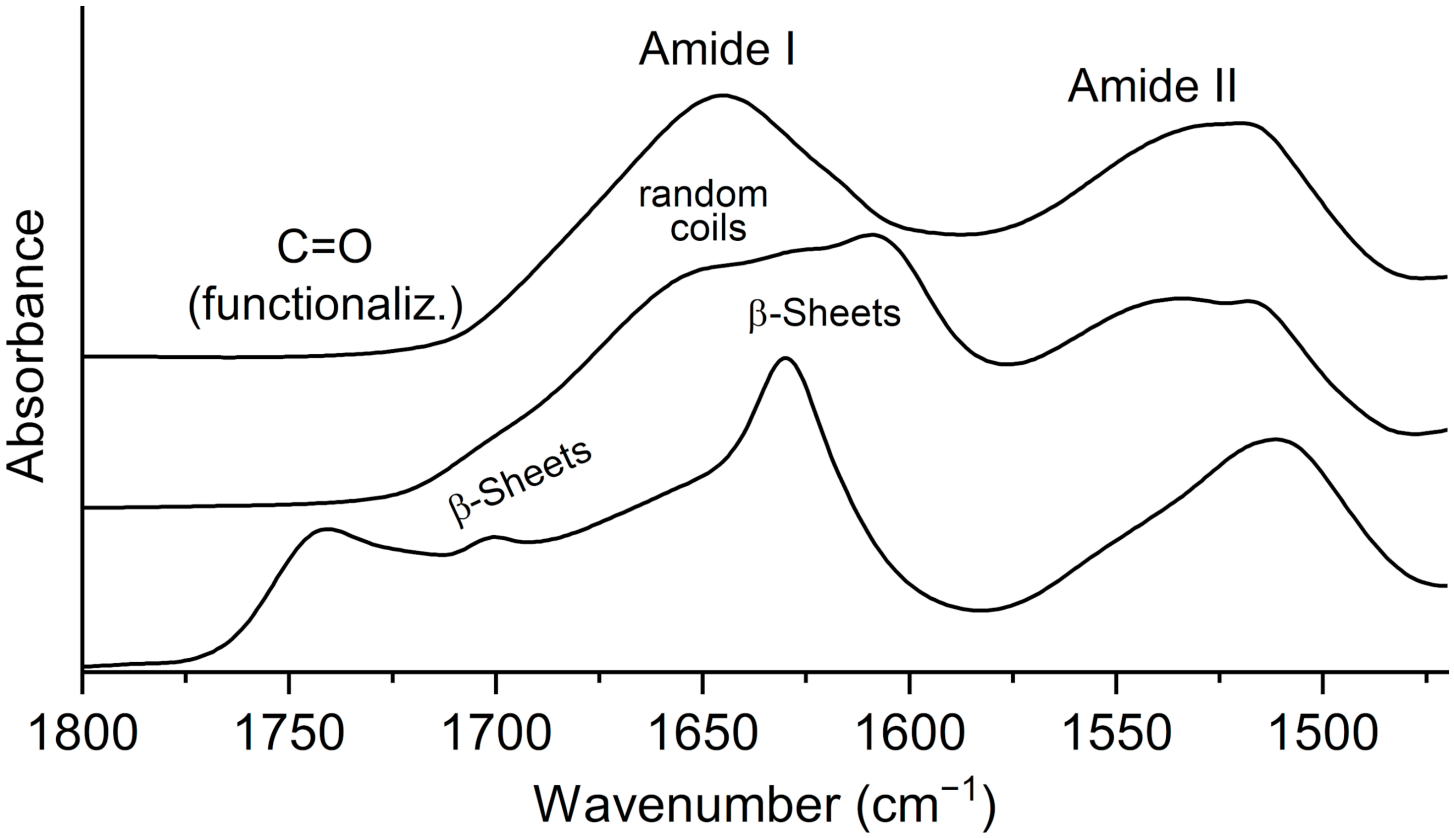
ATR-FTIR absorption spectra of pristine sericin (top), of Ser–reg (middle), and of Ser-Ac (bottom). The main bands and the assignment of Amide I components to the protein secondary structures are indicated.

FTIR spectroscopy was selected for the quantitative characterization of acetylated sericin through the FI and BI, which describe chemical and conformational changes relative to native sericin. The ATR-FTIR spectrum of sericin is shown in Figure 3. As expected, the spectrum is dominated by the characteristic Amide I band (~1700–1600 cm^−1^) and Amide II band (~1600–1500 cm^−1^), reflecting the protein backbone structure [23]. Following acetylation, significant spectral modifications were observed. Most notably, a new absorption band emerged in the 1745–1730 cm^−1^ region, assigned to ester carbonyl (C=O) stretching vibrations. This assignment is supported by the absence of this band in both native and regenerated sericin (Ser-reg; sericin dissolved in DMA/LiCl and subsequently regenerated without acetylation), and its appearance exclusively after reaction with AcCl. Since proteins do not exhibit Amide I absorption in this spectral region, the band provides a suitable marker for monitoring ester formation during acetylation. In order to avoid misinterpretation arising from the presence of residual and/or adsorbed species, the samples were thoroughly washed with water and acetone prior to analysis.

This assignment is further supported by the characteristics of the extracted sericin and the post-synthetic purification procedure. Although high-temperature high-pressure (HTHP) extraction may co-extract trace amounts of non-proteinaceous species that could theoretically participate in side reactions, it has been reported to produce sericin with significantly higher purity than alternative degumming methods [33]. Consequently, the acetylation reaction is expected to occur predominantly on the sericin macromolecular backbone. Moreover, the only potential carbonyl-containing reaction by-product, acetic acid, exhibits an absorption band at slightly lower wavenumbers than those of aliphatic ester groups. In addition, the post-reaction washing cycles with water and acetone ensured the removal of soluble acetic acid and other low-molecular-weight species. Therefore, the absorption band at approximately 1745 cm^−1^ can be confidently assigned to the C=O stretching vibration of ester groups formed on the sericin backbone.

The 3800–3000 cm^−1^ spectral region is characterized by overlapping O–H and N–H stretching vibrations [23,34], including the Amide A and Amide B bands, making direct interpretation difficult. Nevertheless, vector-normalized spectra revealed that samples exhibiting higher ester carbonyl absorption at approximately 1740 cm^−1^ generally showed lower integrated absorption within the 3800–3000 cm^−1^ region. The integrated areas of these two spectral regions exhibited a moderate inverse correlation (Pearson coefficient = −0.64), consistent with partial consumption of hydroxyl and amino functionalities associated primarily with serine and threonine residues during acetylation (Figure S3).

To evaluate whether dissolution in DMA/LiCl could affect the sericin protein structure, the FTIR spectrum of Ser-reg was acquired (Figure 3). The characteristic Amide I and Amide II bands were retained after regeneration, confirming preservation of the protein backbone and indicating that sericin maintained its fundamental protein nature despite some changes in band profile associated with structural reorganization. Together, these qualitative observations demonstrate that (i) acetyl functionalities were successfully introduced onto sericin and (ii) dissolution and regeneration did not compromise the structural integrity of the protein. The FTIR spectra were subsequently subjected to Gaussian deconvolution to quantify the FI and BI, which served as the response variables for the DoE analysis.

### 3.4. FTIR Data Treatment and Deconvolution

**Figure 4:**
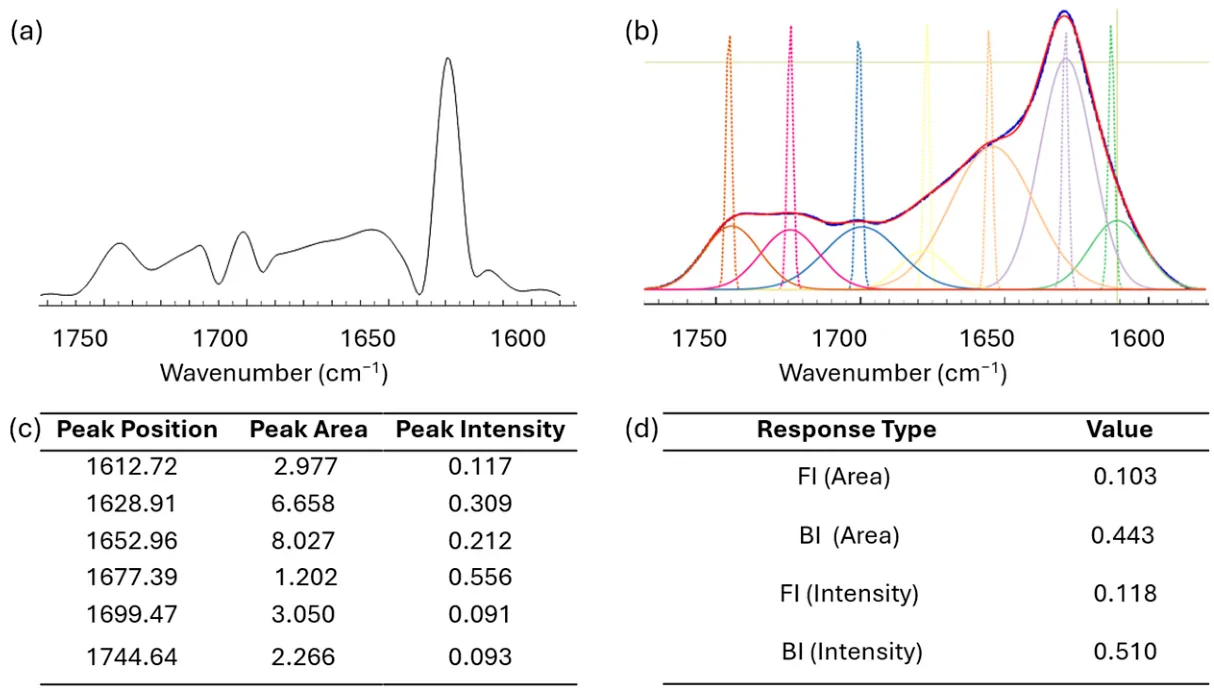
Representative workflow of FTIR data processing in orange for the evaluation of sericin functionalization and secondary structure. (**a**) Inverted second-derivative spectrum used to identify component peaks; (**b**) Gaussian deconvolution of the absorption spectrum, showing the experimental spectrum (dark blue), the overall fitted spectrum (red), and the individual Gaussian components (different colors); (**c**) extracted peak positions, areas, and calculated amplitudes from the fitted components; (**d**) calculated FI and BI based on peak area analysis. The spectrum shown is representative and used for illustrative purposes only.

The FTIR spectra were processed using Orange data mining software [19], and the Amide I region was analyzed through Gaussian peak fitting [14]. The peak areas and corresponding intensities obtained from the fitted components were subsequently used to calculate the response variables employed in the DoE analysis. A representative example of the spectral treatment and the corresponding deconvolution output is shown in Figure 4. It is provided for illustrative purposes and does not correspond to a specific experimental run included in the DoE matrix.

As illustrated, the FTIR data treatment involved several sequential steps. First, the second derivative of the FTIR spectrum was calculated to enhance peak resolution and facilitate the identification of individual component bands [14]. The spectrum was then restricted to the carbonyl spectral region, including the Amide I band (1780–1580 cm^−1^), and inverted to more clearly determine the positions of the derivative peaks, which appear sharper and allow more accurate band localization. The minima identified in the second-derivative spectrum were used as the initial peak-center estimates for the Gaussian deconvolution procedure.

The same preprocessing workflow, fitting strategy, and band-assignment criteria were applied to all spectra throughout the study. Derivative-guided constrained fitting minimized operator-dependent variability while allowing small variations in peak position arising from genuine spectral differences between samples.

To further assess the robustness and reproducibility of the FTIR deconvolution workflow, a representative spectrum was independently fitted five times using the same preprocessing and fitting strategy. The resulting FI and BI descriptors exhibited relative standard deviations of 16.5% (FI area), 11.2% (FI intensity), 9.1% (BI area), and 3.2% (BI intensity), while the reduced χ^2^ values showed an RSD of 5.4% (Table S3). Lower variability was observed for the β-sheet descriptors, whereas the functionalization descriptors showed greater sensitivity to small variations in peak fitting, particularly when area-based calculations were employed. These results demonstrate the high repeatability of the proposed deconvolution procedure, particularly for the BI descriptors, thereby supporting the robustness of the FTIR-based workflow and the reliability of the corresponding response surface models. The comparatively higher variability of the FI descriptors further supports their interpretation as qualitative indicators of the acetylation process rather than robust predictive optimization models. Overall, the low variability observed across repeated fittings indicates limited operator dependence and good reproducibility of the proposed methodology.

Complete deconvolution parameters for all fitted spectra, including the corresponding reduced χ^2^ values, together with the reproducibility assessment, are provided in the Supplementary Materials (Tables S1–S3).

### 3.5. DoE Study of Sericin Acetylation

#### 3.5.1. Exploratory Screening (FFD)

A two-level full factorial design was first employed as an exploratory screening study to identify the experimental variables most likely to influence functionalization index (FI) and structural evolution (BI), thereby guiding the subsequent optimization. Because the design was saturated and provided no residual degrees of freedom, the results are interpreted qualitatively in terms of factor effects rather than formal statistical significance. The experimental design matrix and corresponding responses are summarized in Table 2.

The estimated regression coefficients for both the FI and BI, calculated using peak area and peak intensity, are summarized in Table 3. For the FI, AcCl equivalents exhibited the largest positive contribution, followed by sericin concentration, consistently for both area- and intensity-based descriptors. In contrast, LiCl showed only a minor direct effect within the investigated range, although its influence appeared to depend on its interaction with the other investigated factors. Interaction effects were generally smaller and less consistent between the two descriptors.

Similar qualitative trends were observed for the BI. Sericin concentration and AcCl equivalents exhibited positive coefficients, suggesting that higher levels of these factors favored β-sheet formation, whereas LiCl contributed only marginally within the investigated range. Interaction effects were generally negative or negligible, indicating limited synergistic behavior among the investigated variables.

#### 3.5.2. Optimization Study (BBD)

Following the exploratory screening study, a BBD investigation was performed to model the response surface, evaluate quadratic and interaction effects, and identify the effect of the operating conditions within the investigated experimental domain. Its purpose was not to investigate all possible higher-order interactions, but rather to efficiently estimate the second-order polynomial describing the experimental domain. Although AcCl equivalents and sericin concentration emerged as the most influential variables during the exploratory phase, all three factors were retained for the BBD. While LiCl exhibited only a limited direct effect within the investigated range, it remained essential for protein solubilization and mass transfer. Consequently, all three variables were retained for the response surface optimization.

The experimental design consisted of 15 runs, including three replicated center points (entries O8, O12, and O14; Table 4), which provided an estimate of the pure experimental error for model fitting and enabled the evaluation of model adequacy through the lack-of-fit analysis.

As in the screening phase, two response variables were considered: the FI and the BI, both derived from FTIR spectral deconvolution. These responses were selected to capture both the chemical modification of sericin and the associated structural changes in the protein backbone. The BBD experimental matrix, together with the corresponding response values, is reported in Table 4. Each response was analyzed independently using a second-order response surface model. The corresponding results are presented in the following sections.

##### Functionalization Index

The extent of sericin functionalization was quantitatively evaluated through the FI, defined as the ratio between the ester carbonyl and Amide I bands in the FTIR spectra. FI was calculated using both peak area and peak intensity to compare integrated and amplitude-based descriptors. The experimental data were fitted using second-order quadratic polynomial models, and the estimated regression coefficients together with their statistical significance are reported in Table 5.

For both models, AcCl equivalents were identified as the dominant factor governing functionalization, exhibiting a positive and statistically significant effect (*p* < 0.05). LiCl showed a weaker positive contribution, reaching statistical significance only for the intensity-based model, whereas sericin concentration had no significant linear effect within the investigated range. Among the higher-order terms, the quadratic effect of AcCl^2^ was significant for the intensity-based model, indicating non-linear behavior, while Ser^2^ was also significant only for the intensity-based response. None of the interaction terms reached statistical significance at the 95% confidence level, although the Ser × AcCl interaction approached significance (*p* ≈ 0.06), suggesting a possible antagonistic tendency.

A careful analysis of the center-point replicates (O8, O12, and O14) revealed substantial variability in the FI response. The FI (area) ranged from 0.0940 (O8) to 0.1132 (O14), corresponding to a variation exceeding 20%. This variability may partly reflect the intrinsic heterogeneity and thermal fragmentation characteristic of HTHP-extracted sericin, as evidenced by the SEC and SDS-PAGE analyses. In naturally derived biomaterials, batch-to-batch compositional variability and the coexistence of peptide chains with different molecular weights can reasonably contribute to variability in the measured acetylation response, representing an intrinsic characteristic of the material rather than solely methodological instability.

The quality of the fitted models was assessed using the coefficient of determination (R^2^) and the adjusted coefficient of determination (R^2^adj). The models yielded R^2^ values of 0.851 and 0.917 for the area- and intensity-based FI, respectively, whereas the corresponding adjusted R^2^ values were considerably lower (0.583 and 0.766), particularly for the area-based model. Diagnostic plots (Figures S5 and S6) showed good agreement between observed and predicted values, randomly distributed residuals, and no evidence of systematic deviations or influential outliers, while the coefficient plots (Figure S7) complement the statistical analysis summarized in Table 5. Overall, these results indicate that, although the models describe the general response trends, they do not fully capture the variability of the FI within the investigated experimental domain, making it unsuitable for model validation.

##### β-Sheet Index

The second response evaluated during the optimization study was the BI, which reflects the relative proportion of β-sheet structures within the Amide I region of the FTIR spectra and was used to assess the structural organization of sericin under the investigated reaction conditions. As for the FI, BI was calculated using both peak area and peak intensity, enabling comparison between integrated and direct spectral descriptors (Table 6).

Contrary to the observations for the functionalization Index model, which lacked predictive significance, the regression model exhibits excellent goodness of fit, with R^2^ values of 0.977 and 0.978 for area- and intensity-based BI, respectively. The corresponding adjusted R^2^ values (0.936 and 0.938) remain high, indicating that the model retains strong descriptive capability even after accounting for the number of terms. This close agreement between statistical indicators demonstrates the model’s ability to represent the variability of the BI throughout the experimental domain. Model diagnostics further supported the general adequacy of the fitted models. Diagnostic plots (Figures S8 and S9) showed good agreement between observed and predicted values, randomly distributed residuals, and no evidence of systematic deviations or influential outliers. The coefficient plots (Figure S10) provide a visual representation of the estimated regression coefficients and their associated confidence intervals.

The BI was governed by a complex interplay of linear, interaction, and quadratic effects. Among the linear terms, AcCl equivalents and LiCl concentration exhibited significant negative effects in both models, indicating that increasing either parameter reduced the β-sheet content within the investigated domain. In contrast, the linear effect of sericin concentration was negligible in the area-based model but became significant in the intensity-based model. The Ser × LiCl interaction was significant in both models, whereas the Ser × AcCl interaction was significant only for the intensity-based model. Furthermore, the significant positive quadratic terms for AcCl^2^, Ser^2^, and LiCl^2^ confirm pronounced curvature in the response surface, which ends in a minimum point, indicating that the β-sheet response is governed by non-linear relationships rather than simple linear trends.

**Figure 5:**
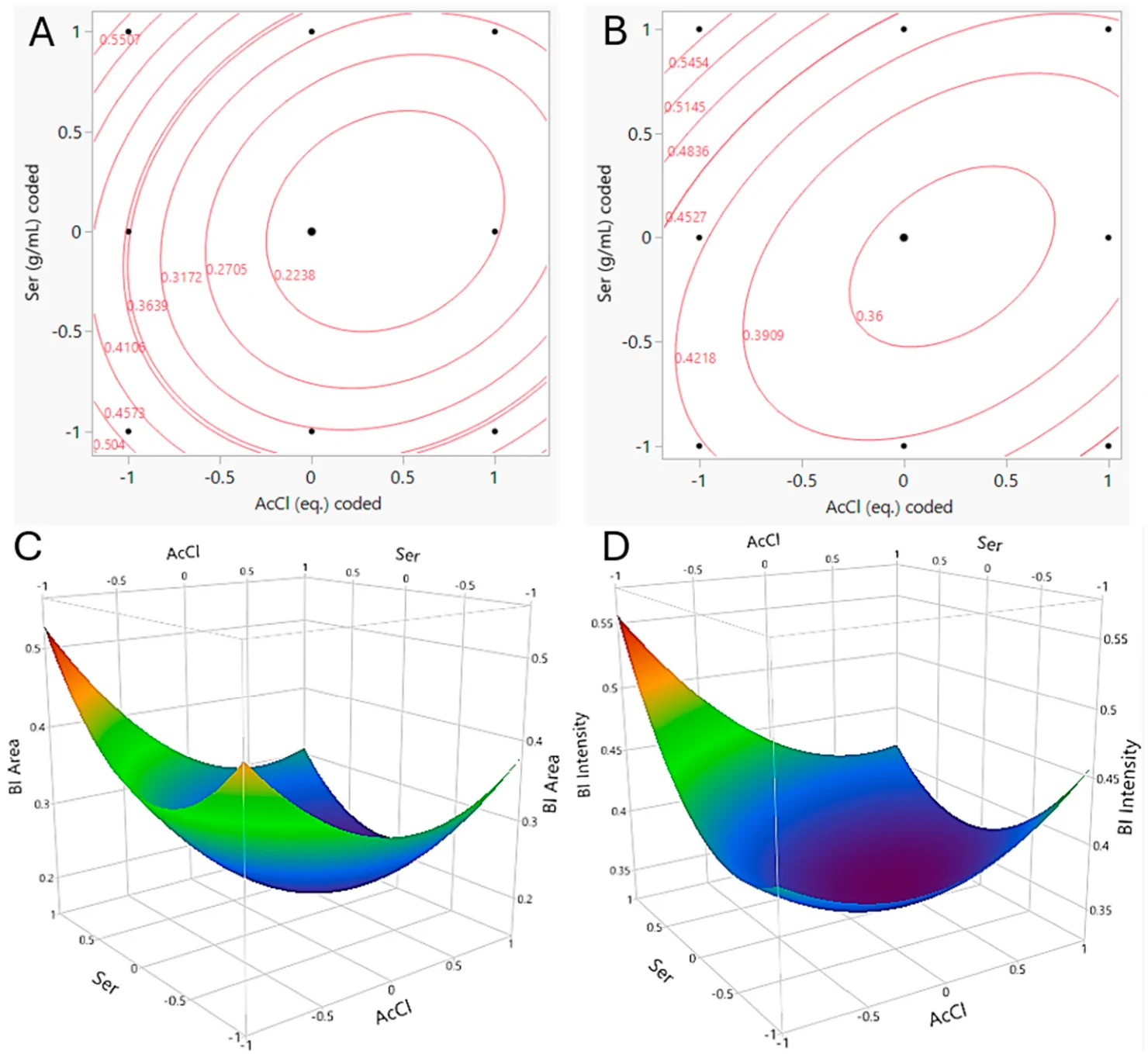
Contour (**A**,**B**) and three-dimensional response surface plots (**C**,**D**) illustrating the effect of AcCl equivalents and sericin concentration on the β-sheet index (BI), with LiCl fixed at its central level. Panels (**A**,**B**) show the contour plots derived from the area- and intensity-based BI models, respectively, with the experimental design points overlaid. Panels (**C**,**D**) present the corresponding three-dimensional response surfaces. The color gradient represents the magnitude of the model-predicted β-sheet Index (BI), with different colors corresponding to different predicted response values. The response surfaces visualize the model-predicted BI across the investigated experimental domain and identify the experimental conditions associated with maximizing the β-sheet Index, highlighting the combined influence of the investigated factors and the nonlinear response behavior.

This behavior is further illustrated by the contour and three-dimensional response surface plots (Figure 5), which visualize the model-predicted BI response across the investigated experimental domain and identify the experimental conditions associated with maximizing the β-sheet Index. As shown in Figure 5, lower BI values are predicted in the central region, whereas increasing values are observed toward the edges of the experimental domain. These trends indicate that β-sheet formation is not linearly dependent on the investigated variables but results from a balance between competing effects related to solvation, reagent concentration, and protein–protein interactions.

From a physicochemical perspective, the observed trends can be rationalized by the combined effects of acetylation and LiCl on protein organization. Acetylation modifies polar protic groups, altering hydrogen-bonding interactions and chain organization, whereas LiCl governs the balance between chain solvation and intermolecular association. Higher LiCl concentrations promote a more solvated and less associated state, while lower concentrations favor chain proximity and β-sheet organization. Similarly, increasing AcCl equivalents may introduce structural perturbations that limit further organization while promoting chemical functionalization. Consequently, the β-sheet content depends on the combined influence of chemical modification, solvent composition, and protein concentration rather than on any single reaction parameter. The observed trends therefore reflect a redistribution of structural organization across the investigated experimental space rather than a direct simple interplay between functionalization and β-sheet formation.

Taken together, the FI and BI analyses demonstrate that sericin acetylation is governed by a multivariable interplay between reaction chemistry and protein structural organization. Whereas FI is primarily controlled by AcCl availability, BI depends on a more complex combination of reagent concentration, protein concentration, solvent composition, and their interactions. Although both area- and intensity-based descriptors exhibited consistent overall trends, minor differences between them are expected because they describe different characteristics of the fitted Gaussian bands. The intensity-based descriptors are primarily influenced by the maximum absorbance of the fitted component, whereas the area-based descriptors additionally reflect variations in band width introduced during the deconvolution procedure. Consequently, the FI descriptors, which rely on the ester carbonyl contribution, are inherently more sensitive to small fitting variations and local chemical heterogeneity, consistent with the higher variability observed in the repeatability analysis (Table S3). In contrast, the BI descriptors exhibited substantially higher reproducibility and stronger statistical performance, supporting their use as the primary descriptors for interpreting the structural evolution of sericin, while the FI descriptors are best regarded as qualitative indicators of the acetylation process. Although clear correlations between functionalization and structural organization were observed, the present methodology does not establish a direct causal relationship between acetylation and β-sheet evolution. Instead, the results indicate that the reaction conditions can be systematically tuned to modulate both chemical functionalization and protein secondary structure within the explored design space.

##### Validation of the Predictive Model

Model validation for the BI response was carried out by testing five independent validation experiments that were not coincident with the experimental points used for model development and comparing the experimental responses with the corresponding model predictions and their associated 95% prediction intervals. Prediction intervals were selected because they account for both model uncertainty and experimental variability associated with new observations.

For all validation conditions, the experimental BI values remained within the corresponding 95% prediction intervals of the model predictions (Table 7). Although some deviations between predicted and experimental values were observed, particularly for V2, V3, and V5, these remained within the expected predictive uncertainty of the model.

Overall, the validation results support the predictive capability of the developed BI models within the investigated experimental domain. Although five independent validation experiments provided an additional assessment of model performance, further validation across a broader range of experimental conditions would strengthen confidence in the general applicability of the proposed models.

## 4. Conclusions

This study systematically investigated the acetylation of sericin in a DMA/LiCl solvent system using a Design of Experiments (DoE) approach combined with quantitative FTIR analysis. Acetylation was selected as a model functionalization reaction to evaluate the influence of acetyl chloride (AcCl) equivalents, sericin concentration, and LiCl concentration on both the extent of chemical modification and protein secondary structure.

A quantitative FTIR workflow based on spectral deconvolution was established to monitor the reaction through two complementary response variables: the functionalization index (FI), describing the relative extent of acetylation, and the β-sheet index (BI), reflecting changes in protein secondary structure. The exploratory screening identified AcCl as the dominant factor influencing functionalization. Subsequent response surface optimization showed that the FI models captured the general trends of the system but exhibited limited descriptive capability after accounting for model complexity. In contrast, the BI was successfully described by robust quadratic models (R^2^ = 0.977–0.978; adjusted R^2^ = 0.936–0.938), revealing significant linear, interaction, and quadratic effects. In particular, AcCl and LiCl exhibited significant negative linear effects on β-sheet content, while the significant interaction and quadratic terms demonstrated that structural organization depends on a complex interplay between reagent concentration, protein concentration, and solvent composition.

The proposed FTIR-based methodology was further supported by several complementary observations. Qualitative spectral analysis confirmed the formation of ester functionalities through the appearance of the characteristic carbonyl absorption band, while the moderate inverse correlation between the ester carbonyl and hydroxyl stretching regions (Pearson coefficient = −0.64) was consistent with the progressive consumption of reactive hydroxyl groups during acetylation. Moreover, the reproducibility assessment demonstrated limited operator dependence of the deconvolution workflow, and independent validation experiments supported the predictive capability of the developed response surface models within the investigated experimental domain. Together, these findings reinforce the robustness of the proposed analytical approach for quantitatively investigating sericin functionalization.

These findings indicate that the reaction conditions influence not only the extent of acetylation but also the structural organization of sericin. The observed changes in β-sheet content suggest that protein conformation evolves in response to the reaction environment, highlighting the importance of considering structural effects alongside chemical modification when designing functionalization strategies for natural proteins.

Overall, this work demonstrates that combining quantitative FTIR analysis with DoE provides a systematic framework for investigating sericin functionalization and its associated structural evolution. Although the present study was not intended to evaluate material performance directly, the proposed methodology establishes a quantitative basis for future investigations aimed at correlating reaction conditions with protein structure and physicochemical properties. Consequently, this approach may support the rational development and optimization of chemically modified sericin for future applications in biomaterials, coatings, composite materials, and subsequent chemical functionalization.

## Figures and Tables

**Table 1:** Coded and actual factor levels used in the screening (A, two-level full factorial design) and optimization (B, Box–Behnken design) phases for sericin acetylation. The investigated factors include sericin concentration (g/mL), acetyl chloride equivalents (AcCl, equiv.), and LiCl concentration in DMA (*w*/*v* %).

**(A) Sericin (g/mL)**			**AcCl (Equiv.)**				**LiCl in DMA (*w*/*v* %)**		
−1		+1	−1		+1		−1	+1	
0.01		0.05	5		50		1	5	
**(B) Sericin (g/mL)**			**AcCl (Equiv.)**				**LiCl in DMA (*w*/*v* %)**		
−1	0	+1	−1	0		+1	−1	0	+1
0.01	0.03	0.05	5	27.5		50	1	5	10

**Table 2:** Experimental design matrix and corresponding responses obtained during the screening phase. Actual values of the investigated factors, AcCl, equiv., LiCl concentration in DMA (*w*/*v* %), and Ser (g/mL), are reported alongside their coded levels (in parentheses). The responses are expressed as FI and BI, calculated from both peak area and peak intensity (amplitude). The coded levels −1 and +1 correspond to the low and high levels of each factor, respectively.

Run	AcCl (Equiv.)	LiCl in DMA (*w*/*v* %)	Sericin (g/mL)	FI (Area)	BI (Area)	FI (Intensity)	BI (Intensity)
S1	5 (−1)	1 (−1)	0.01 (−1)	0.091	0.379	0.118	0.432
S2	5 (−1)	1 (−1)	0.05 (+1)	0.101	0.542	0.117	0.574
S3	5 (−1)	10 (+1)	0.01 (−1)	0.004	0.435	0.009	0.416
S4	5 (−1)	10 (+1)	0.05 (+1)	0.086	0.514	0.099	0.549
S5	50 (+1)	1 (−1)	0.01 (−1)	0.098	0.521	0.117	0.538
S6	50 (+1)	1 (−1)	0.05 (+1)	0.121	0.516	0.133	0.553
S7	50 (+1)	10 (+1)	0.01 (−1)	0.104	0.517	0.112	0.550
S8	50 (+1)	10 (+1)	0.05 (+1)	0.148	0.425	0.169	0.497

**Table 3:** Estimated regression coefficients obtained from the exploratory screening model for FI and BI, evaluated using both peak area and peak intensity.

Term	Coefficient		Coefficient	
	FI (Area)	BI (Area)	FI (Intensity)	BI (Intensity)
Intercept	0.094	0.481	0.109	0.514
AcCl	0.024	0.013	0.023	0.021
LiCl	−0.009	−0.008	−0.012	−0.010
Ser	0.020	0.018	0.020	0.030
LiCl × AcCl	0.017	−0.015	−0.020	−0.0003
Ser × AcCl	−0.003	−0.042	−0.002	−0.039
Ser × LiCl	0.012	−0.021	0.016	−0.010

**Table 4:** Experimental design and corresponding responses obtained during the optimization phase (Box–Behnken design). Experimental values of the investigated factors are reported together with their coded levels (in parentheses). The response variables are expressed as FI and BI. The coded values −1, 0, and +1 correspond to the low, center, and high levels of each factor, respectively. Central points triplicate are in red.

Run	AcCl (Equiv.)	LiCl in DMA (*w*/*v* %)	Sericin (g/mL)	FI (Area)	BI (Area)	FI (Intensity)	BI (Intensity)
O1	5 (−1)	5 (0)	0.01 (−1)	0.0040	0.4351	0.0089	0.4164
O2	5 (−1)	1 (−1)	0.03 (0)	0.0709	0.5509	0.0880	0.5329
O3	5 (−1)	10 (+1)	0.03 (0)	0.0735	0.4044	0.0907	0.4669
O4	5 (−1)	5 (0)	0.05 (+1)	0.0860	0.5144	0.0991	0.5488
O5	27.5 (0)	1 (−1)	0.01 (−1)	0.0725	0.4240	0.0858	0.4654
O6	27.5 (0)	10 (+1)	0.01 (−1)	0.1597	0.4304	0.1731	0.4572
O7	27.5 (0)	1 (−1)	0.05 (+1)	0.0947	0.5440	0.1031	0.5759
O8	27.5 (0)	5 (0)	0.03 (0)	0.0940	0.1771	0.1430	0.3291
O9	50 (+1)	5 (0)	0.05 (+1)	0.0788	0.3150	0.1095	0.4237
O10	50 (+1)	10 (+1)	0.03 (0)	0.1147	0.2091	0.1711	0.3689
O11	27.5 (0)	10 (+1)	0.05 (+1)	0.0963	0.3322	0.1307	0.4317
O12	27.5 (0)	5 (0)	0.03 (0)	0.1094	0.2077	0.1595	0.3632
O13	50 (+1)	5 (0)	0.01 (−1)	0.1040	0.3919	0.1211	0.4665
O14	27.5 (0)	5 (0)	0.03 (0)	0.1132	0.2192	0.1625	0.3701
O15	50 (+1)	1 (−1)	0.03 (0)	0.1139	0.4298	0.1267	0.4974

**Table 5:** Regression coefficients, standard errors, and associated *p*-values of the Box–Behnken regression models for the functionalization index (FI), evaluated using both peak area and peak intensity (amplitude). The table reports the estimated effects of the main factors (AcCl equivalents, LiCl concentration, and sericin concentration), together with their interaction and quadratic terms.

Term	FI (Area)			FI (Intensity)		
	Coefficient	Std. Error	*p*-Value	Coefficient	Std. Error	*p*-Value
Intercept	0.106	0.012	0.0004 *	0.155	0.012	<0.0001 *
AcCl	0.022	0.008	0.034 *	0.030	0.007	0.009 *
LiCl	0.012	0.008	0.190	0.020	0.007	0.040 *
Ser	0.002	0.008	0.809	0.007	0.007	0.402
LiCl × AcCl	−0.0005	0.011	0.967	0.010	0.010	0.360
Ser × AcCl	−0.027	0.011	0.055	−0.025	0.010	0.057
Ser × LiCl	−0.021	0.011	0.104	−0.015	0.010	0.209
AcCl^2^	−0.025	0.011	0.077	−0.037	0.011	0.018 *
Ser^2^	−0.012	0.011	0.320	−0.033	0.011	0.028 *
LiCl^2^	0.013	0.011	0.310	0.001	0.011	0.910

* Statistically significant (*p* ≤ 0.05).

**Table 6:** Regression coefficients, standard errors, and associated *p*-values obtained from the Box–Behnken optimization models for the β-sheet index (BI), evaluated using both peak area and peak intensity (amplitude). The table reports the estimated effects of the main factors (AcCl equivalents, LiCl concentration, and sericin concentration), together with their interaction and quadratic terms.

Term	BI (Area)			BI (Intensity)		
	Coefficient	Std. Error	*p*-Value	Coefficient	Std. Error	*p*-Value
Intercept	0.201	0.018	0.0001 *	0.354	0.010	<0.0001 *
AcCl	−0.070	0.011	0.002 *	−0.026	0.006	0.009 *
LiCl	−0.072	0.011	0.001 *	−0.043	0.006	0.001 *
Ser	0.003	0.011	0.796	0.022	0.006	0.019 *
LiCl × AcCl	−0.019	0.016	0.291	−0.016	0.009	0.143
Ser × AcCl	−0.040	0.016	0.056	−0.044	0.009	0.005 *
Ser × LiCl	−0.056	0.016	0.018 *	−0.034	0.009	0.013 *
AcCl^2^	0.089	0.016	0.003 *	0.044	0.009	0.004 *
Ser^2^	0.123	0.016	0.0007 *	0.063	0.009	0.001 *
LiCl^2^	0.108	0.016	0.001 *	0.066	0.009	0.001 *

* Statistically significant (*p* ≤ 0.05).

**Table 7:** Experimental conditions and validation of the BI response surface models through comparison of experimental and predicted responses (95% prediction intervals).

Run	AcCl (Equiv.)		LiCl in DMA (*w*/*v* %)	Ser (g/mL)
V1 *	50 (+1)		10 (+1)	0.01 (−1)
V2	20 (−0.33)		3 (−0.5)	0.02 (−0.5)
V3	5 (−1)		10 (+1)	0.05 (+1)
V4 *	50 (+1)		10 (+1)	0.01 (−1)
V5	50 (+1)		10 (+1)	0.05 (+1)
	BI (Area)		BI (Intensity)	
Run	Exp.	Theo.	Exp.	Theo.
V1 *	0.43	0.45 (±0.13)	0.47	0.50 (±0.07)
V2	0.46	0.30 (±0.09)	0.47	0.39 (±0.05)
V3	0.39	0.53 (±0.12)	0.39	0.56 (±0.07)
V4 *	0.42	0.45 (±0.13)	0.42	0.50 (±0.07)
V5	0.44	0.27 (±0.13)	0.44	0.39 (±0.07)

* V1 and V4 correspond to duplicate validation experiments performed under identical reaction conditions to assess experimental reproducibility.

## Data Availability

All data supporting the findings of this study are included in the Supplementary Materials. Additional data related to this study are available from the corresponding author upon reasonable request.

## Supplementary Materials

The following supporting information can be downloaded at https://www.mdpi.com/article/10.3390/polym18161960/s1. Figure S1. Visual assessment of sericin solubility behavior in different solvent systems over time. (A) Snapshots of sericin samples immediately after preparation (t = 0 min), showing the initial dispersion state in the respective solvents. (B) Snapshots after 60 min, illustrating the evolution of the systems, including changes in clarity and the onset of phase separation or sedimentation. (C) Snapshots after 150 min, highlighting the formation of suspensions and/or precipitates, providing a qualitative comparison of solvent effectiveness in maintaining sericin dispersion. Figure S2. Concentration-dependent solubility of sericin in selected solvents. Representative images of samples prepared in water, DMSO, DMA, and tert-butyl acetate at concentrations ranging from 100 to 10 mg/mL after heating at 50 °C for 30 min. The accompanying table summarizes the observed physical states (foam, liquid, suspension, or residue) for each condition. Figure S3. FTIR absorption in the 3800–3000 cm^−1^ and 1800–1600 cm^−1^ spectral regions. (A) FTIR absorption spectra of the functionalized samples. Samples with a low intensity of the band at approximately 1740 cm^−1^ are shown in blue, those with high intensity in red, and those with intermediate intensity in magenta. (B) Scatter plot of the integrated area between 1800 and 1710 cm^−1^ versus the integrated area between 3800 and 3000 cm^−1^ (black squares), together with the corresponding linear regression (red line). To allow a meaningful comparison of the integrated areas, the spectra were first vector normalized using Orange Data Mining (as shown in panel A), after which the band areas were calculated. The scatter plot and Pearson correlation coefficient were generated using OriginPro software. Figure S4. Representative constrained Gaussian deconvolutions of the FTIR Amide I region for samples exhibiting low (Run O1), intermediate (Run O8), and high (Run O15) Functionalization Index (FI) values. The experimental spectra (black) are shown together with the cumulative fitted spectra (red) and the individual Gaussian components. Peak centers were initialized from the second-derivative spectra and fitted using the same constrained fitting strategy described in the Methods. The close agreement between the experimental and fitted spectra illustrates the robustness of the deconvolution procedure across the investigated range of functionalization. Table S1. FTIR deconvolution parameters and calculated response variables obtained for the screening experiments (S1–S8). Peak positions were initialized from the minima of the second-derivative spectra and refined by constrained nonlinear Gaussian fitting, with peak centers allowed to vary within predefined peak-specific intervals (typically ±1 to ±5 cm^−1^) around their initial positions. For each Gaussian component, the fitted center (cm^−1^), area, standard deviation (σ, related to the full width at half maximum), and intensity are reported. Reduced χ^2^ values are provided as goodness-of-fit metrics for the Gaussian deconvolution. FI and BI were calculated using both peak area- and intensity-based descriptors. Table S2. FTIR deconvolution parameters and calculated response variables obtained for the optimization experiments (O1–O15). Peak positions were initialized from the minima of the second-derivative spectra and refined by constrained nonlinear Gaussian fitting, with peak centers allowed to vary within predefined peak-specific intervals (typically ±1 to ±5 cm^−1^) around their initial positions. For each Gaussian component, the fitted center (cm^−1^), area, standard deviation (σ, related to the full width at half maximum), and intensity are reported. Reduced χ^2^ values are provided as goodness-of-fit metrics for the Gaussian deconvolution. FI and BI were calculated using both peak area- and intensity-based descriptors. Table S3. Reproducibility assessment of the FTIR deconvolution workflow based on five independent Gaussian fits of a representative spectrum. Individual fitting parameters, reduced χ^2^ values, and the resulting FI and BI descriptors are reported. Mean, standard deviation (SD), and relative standard deviation (RSD) are provided for the calculated descriptors and reduced χ^2^ values. Figure S5. Diagnostic evaluation of the area-based FI response model. (A) Observed versus predicted values with 95% confidence bands; (B) residuals versus predicted values; (C) studentized residuals versus run order; (D) lack-of-fit analysis; and (E) analysis of variance (ANOVA). The model showed good agreement between observed and predicted responses (R^2^ = 0.85, RMSE = 0.0215), while the residuals were randomly distributed around zero with no evident systematic trend, supporting the adequacy of the fitted model. The lack-of-fit test was not statistically significant (*p* = 0.1303), indicating that the quadratic model adequately described the experimental data within the investigated design space. Although the overall model was not statistically significant (ANOVA, *p* = 0.1078), this result is consistent with the exploratory nature of the FI response discussed in the manuscript. Figure S6. Diagnostic evaluation of the intensity-based FI response model. (A) Observed versus predicted values with 95% confidence bands; (B) residuals versus predicted values; (C) studentized residuals versus run order; (D) lack-of-fit analysis; and (E) analysis of variance (ANOVA). The model showed good agreement between observed and predicted responses (R^2^ = 0.92, RMSE = 0.0207), while the residuals were randomly distributed around zero with no evident systematic trend, supporting the adequacy of the fitted model. The lack-of-fit test was not statistically significant (*p* = 0.1498), indicating that the quadratic model adequately described the experimental data within the investigated design space. The overall model was statistically significant (ANOVA, *p* = 0.0303), supporting the reliability of the fitted regression model. Figure S7. Coefficient plots illustrating the estimated effects of model terms on the FI obtained from the Box–Behnken design. (A) FI calculated from peak area and (B) FI calculated from peak intensity (amplitude). Error bars represent confidence intervals, and positive and negative coefficients indicate enhancing or reducing contributions to the FI response, respectively. Figure S8. Diagnostic evaluation of the area-based BI response model. (A) Observed versus predicted values with 95% confidence bands; (B) residuals versus predicted values; (C) studentized residuals versus run order; (D) lack-of-fit analysis; and (E) analysis of variance (ANOVA). The model showed excellent agreement between observed and predicted responses (R^2^ = 0.98, RMSE = 0.0014), while the residuals were randomly distributed around zero with no evident systematic trend, supporting the adequacy of the fitted model. The lack-of-fit test was not statistically significant (*p* = 0.2735), indicating that the quadratic model adequately described the experimental data within the investigated design space. The overall model was highly statistically significant (ANOVA, *p* = 0.0014), confirming the robustness and predictive capability of the fitted regression model. Figure S9. Diagnostic evaluation of the intensity-based BI response model. (A) Observed versus predicted values with 95% confidence bands; (B) residuals versus predicted values; (C) studentized residuals versus run order; (D) lack-of-fit analysis; and (E) analysis of variance (ANOVA). The model showed excellent agreement between observed and predicted responses (R^2^ = 0.98, RMSE = 0.0018), while the residuals were randomly distributed around zero with no evident systematic trend, supporting the adequacy of the fitted model. The lack-of-fit test was not statistically significant (*p* = 0.7438), indicating that the quadratic model adequately described the experimental data within the investigated design space. The overall model was highly statistically significant (ANOVA, *p* = 0.0013), confirming the robustness and predictive capability of the fitted regression model. Figure S10. Coefficient plots illustrating the estimated effects of model terms on the BI obtained from the Box–Behnken design. (A) Results based on BI calculated from peak area and (B) results based on BI calculated from peak intensity (amplitude). Error bars represent confidence intervals, and positive and negative coefficients indicate enhancing or reducing contributions to the BI response, respectively.
