# Supplementary material for: Design of Experiments Investigation of Sericin Acetylation Using a Quantitative FTIR Approach
Source: Polymers (Basel). 2026 Aug 11;18(16):1960. doi: 10.3390/polym18161960 (PMC13516617; doi:10.3390/polym18161960)
Supplement: Supplementary file 1 [file polymers-18-01960-s001.zip › polymers-4459648-supplementary.pdf]

Supplementary Information

Design of Experiments Investigation of Sericin Acetylation Using a Quantitative FTIR Approach

Rony Aad [1], Luca Leuzzi [2,†], Diletta Ami [2], Greta Bianchi [2], Marco Mangiagalli [2], Antonino Natalello [2], Laura Cipolla [2,*] and Simone Vesentini [1,*]

[1] Department of Electronics, Information, and Bioengineering, Politecnico di Milano, 20133 Milano, Italy; rony.aad@polimi.it
[2] Department of Biotechnology and Biosciences, University of Milano-Bicocca, 20126 Milano, Italy; luca.leuzzi@unimib.it (L.L.); diletta.ami@unimib.it (D.A.); greta.bianchi@unimib.it (G.B.); marco.mangiagalli@unimib.it (M.M.); antonino.natalello@unimib.it (A.N.)
* Correspondence: laura.cipolla@unimib.it (L.C.); simone.vesentini@polimi.it (S.V.)
† Current address: Department of Chemistry, Materials and Chemical Engineering "Giulio Natta", Politecnico di Milano, 20133 Milano, Italy.

S1. Solubility Studies 2
S2. FTIR – Characterization of the 3800–3000 cm^{-1} spectral region 4
S3. DoE study of sericin acetylation 4
S3.1. *Calculation of experimental reagent and solvent amounts* 5
S3.2. *Representative Gaussian deconvolutions* 6
S3.3. *Screening Phase – Full Factorial Design (FFD)* 7
S3.4. *Optimization Phase – Box-Behnken Design (BBD)* 8

S1. Solubility Studies

Preliminary qualitative screening of sericin solubility was performed at a concentration of 1 mg/mL using a range of solvents, including water (01), dimethyl sulfoxide (DMSO) (02), *N,N*-dimethylformamide (DMF) (03), *N,N*-dimethylacetamide (DMA) (04), 1,4-dioxane (05), tert-butyl acetate (t-BuOAc) (06), triethylamine (TEA) (07), tetrahydrofuran (THF) (08), acetonitrile (MeCN) (09), and a THF:MeCN (1:1, v/v) mixture (10). The numbering corresponds to the order of the samples shown from left to right in Figure S1. The solubility behavior was visually monitored over time at t = 0, 60, and 150 min to assess dispersion stability, clarity, and the formation of suspensions or precipitates.

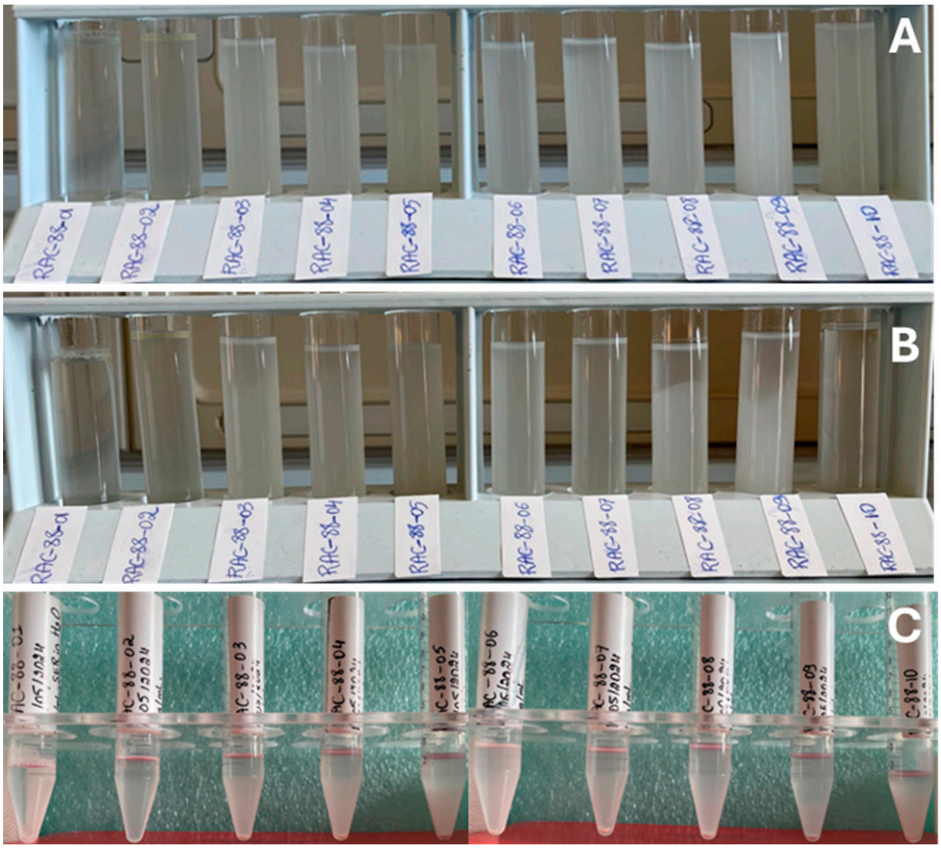

Figure S1. Visual assessment of sericin solubility behavior in different solvent systems over time. (A) Snapshots of sericin samples immediately after preparation (t = 0 min), showing the initial dispersion state in the respective solvents. (B) Snapshots after 60 min, illustrating the evolution of the systems, including changes in clarity and the onset of phase separation or sedimentation. (C) Snapshots after 150 min, highlighting the formation of suspensions and/or precipitates, providing a qualitative comparison of solvent effectiveness in maintaining sericin dispersion.

The concentration-dependent solubility of sericin was further investigated in four selected solvents, water, DMSO, DMA, and t-BuOAc, to identify conditions enabling homogeneous solutions at higher concentrations. Serial dilutions from 100 to 10 mg/mL were prepared under heating at 50 °C for 30 min. The resulting systems were visually evaluated in terms of clarity, phase behavior, and the presence of foam, suspensions, or residues. The observed physical states for each concentration and solvent and the representative images of the corresponding samples are shown in Figure S2.

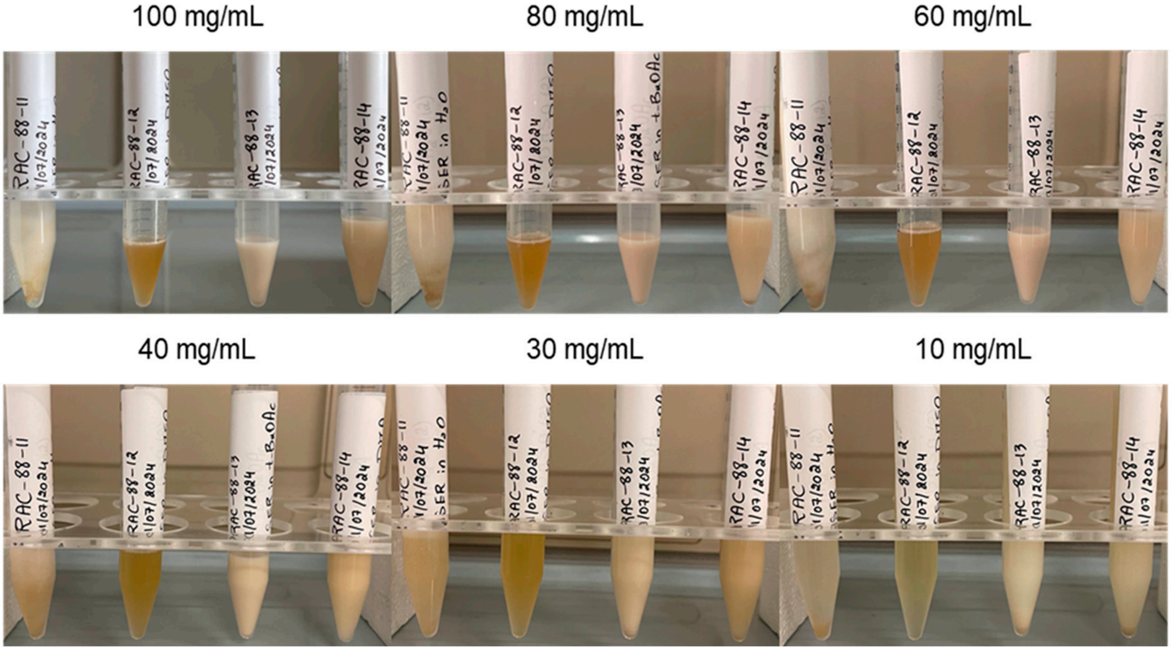

Solvent	100 mg/mL	90 mg/mL	80 mg/mL	60 mg/mL	40 mg/mL	30 mg/mL	10 mg/mL
**H_2O**	Foam	Foam	Foam	Foam	Foam/liquid	Liquid/suspension	Clear liquid/residue
**DMSO**	Dark yellow liquid	Dark yellow liquid	Dark yellow liquid	Dark yellow liquid	Clearer yellow liquid	Clearer yellowish liquid	Clearer lighter liquid
**t-BuOAc**	Suspension	Suspension	Suspension	Suspension	Suspension	Suspension	Suspension/residue
**DMA**	Suspension	Suspension	Suspension	Suspension	Suspension	Suspension	Suspension/residue

Figure S2. Concentration-dependent solubility of sericin in selected solvents. Representative images of samples prepared in water, DMSO, DMA, and tert-butyl acetate at concentrations ranging from 100 to 10 mg/mL after heating at 50 °C for 30 min. The accompanying table summarizes the observed physical states (foam, liquid, suspension, or residue) for each condition.

S2. FTIR – Characterization of the 3800–3000 cm⁻¹ spectral region

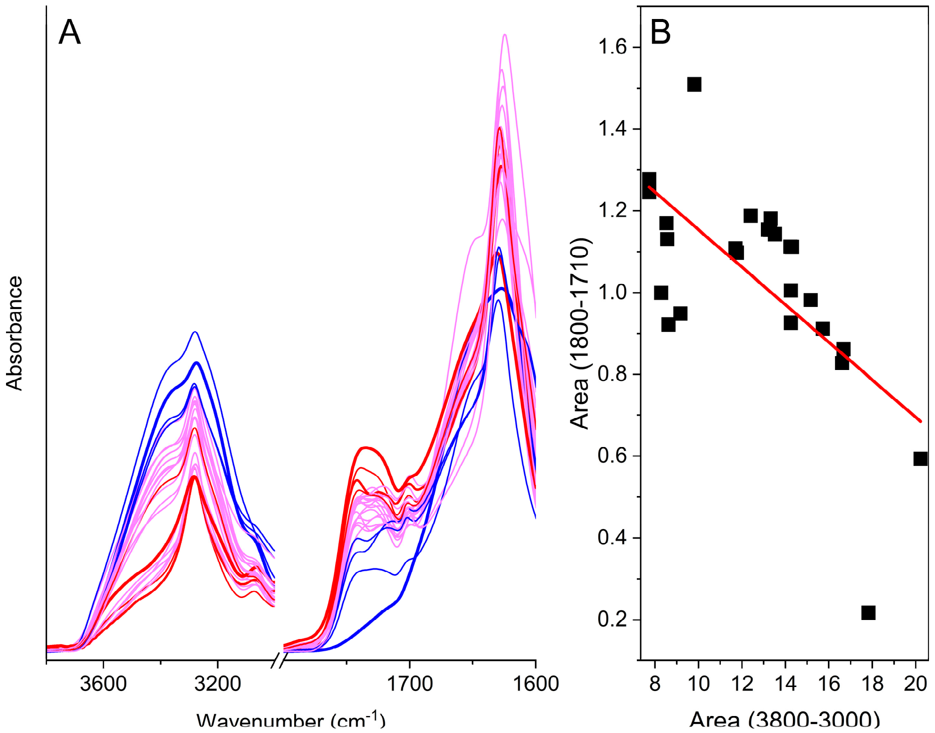

Figure S3. FTIR absorption in the 3800–3000 cm^{-1} and 1800–1600 cm^{-1} spectral regions. (A) FTIR absorption spectra of the functionalized samples. Samples with a low intensity of the band at approximately 1740 cm^{-1} are shown in blue, those with high intensity in red, and those with intermediate intensity in magenta. (B) Scatter plot of the integrated area between 1800 and 1710 cm^{-1} versus the integrated area between 3800 and 3000 cm^{-1} (black squares), together with the corresponding linear regression (red line). To allow a meaningful comparison of the integrated areas, the spectra were first vector normalized using Orange Data Mining (as shown in panel A), after which the band areas were calculated. The scatter plot and Pearson correlation coefficient were generated using OriginPro software.

S3. DoE study of sericin acetylation

To define the experimental matrix for the Design of Experiments (DoE), the lower and upper limits of the selected factors were first established based on preliminary feasibility considerations. In the screening phase, the investigated variables were sericin concentration in DMA, acetyl chloride equivalents, and LiCl concentration in DMA. For consistency across all experiments, the mass of sericin was fixed at 0.5 g, and the corresponding quantities of solvent, reagent, and salt were calculated accordingly. Since sericin concentration was treated as a factor in the design, the volume of DMA was adjusted according to the selected concentration level. The amount of acetyl chloride was determined based on the estimated molar amount of reactive nucleophilic groups in sericin, while the amount of LiCl was calculated as a function of the corresponding solvent volume and selected w/v concentration. The calculations used to derive the actual values employed in the experimental runs are detailed below.

S3.1. Calculation of experimental reagent and solvent amounts

Actual quantities of DMA, AcCl, and LiCl were calculated from the selected factor levels using a fixed sericin mass (0.5 g). Considering that approximately 33% of sericin's amino acid composition consists of nucleophilic residues, mainly associated with serine, threonine, and lysine, the mass of potentially reactive groups was estimated as:

$$0.5 \times 0.33 = 0.165\, g$$

Using an average residue molecular weight of 105.45 g/mol, the molar amount of reactive groups was estimated as:

$$n = \frac{0.165}{105.45} = 1.57 \times 10^{-3}\, mol$$

This value was used to calculate AcCl equivalents. DMA volume was calculated from the selected sericin concentration:

$$V_{DMA} = \frac{m_{sericin}}{C_{sericin}}$$

Accordingly, the calculated DMA volumes were 50 mL, 16.67 mL, and 10 mL for sericin concentrations of 0.01, 0.03, and 0.05 g/mL, respectively.

The amount of AcCl was calculated based on the selected equivalents relative to the estimated 1.57 mmol of reactive groups:

$$n_{AcCl} = Eq. \times 1.57 mmol$$

AcCl volume was calculated using MW = 78.50 g/mol and ϱ = 1.104 g/mL:

$$V_{AcCl} = \frac{n \times MW}{\rho}$$

Accordingly, the calculated AcCl volumes were 0.56 mL, 3.0 mL, and 5.6 mL for 5, 27.5, and 50 equivalents, respectively.

LiCl was added according to the selected concentration (w/v %) relative to DMA volume. The mass of DMA was calculated from its corresponding volume using the density of DMA, according to:

$$LiCl(g) = \frac{C_{LiCl}}{100} \times V_{DMA}$$

where C_{LiCl} is the LiCl concentration expressed as w/v %.

Accordingly, the amount of LiCl depended on both the DMA volume and the selected LiCl concentration, resulting in three different values for each solvent volume corresponding to 1, 5, and 10% (w/v). Thus, for a DMA volume of 50 mL, the required LiCl amounts were 0.50 g, 2.50 g, and 5.00 g, respectively. For 16.67 mL of DMA, the corresponding values were 0.17 g, 0.83 g, and 1.67 g, while for 10 mL of DMA they were 0.10 g, 0.50 g, and 1.00 g, respectively.

S3.2. Representative Gaussian deconvolutions

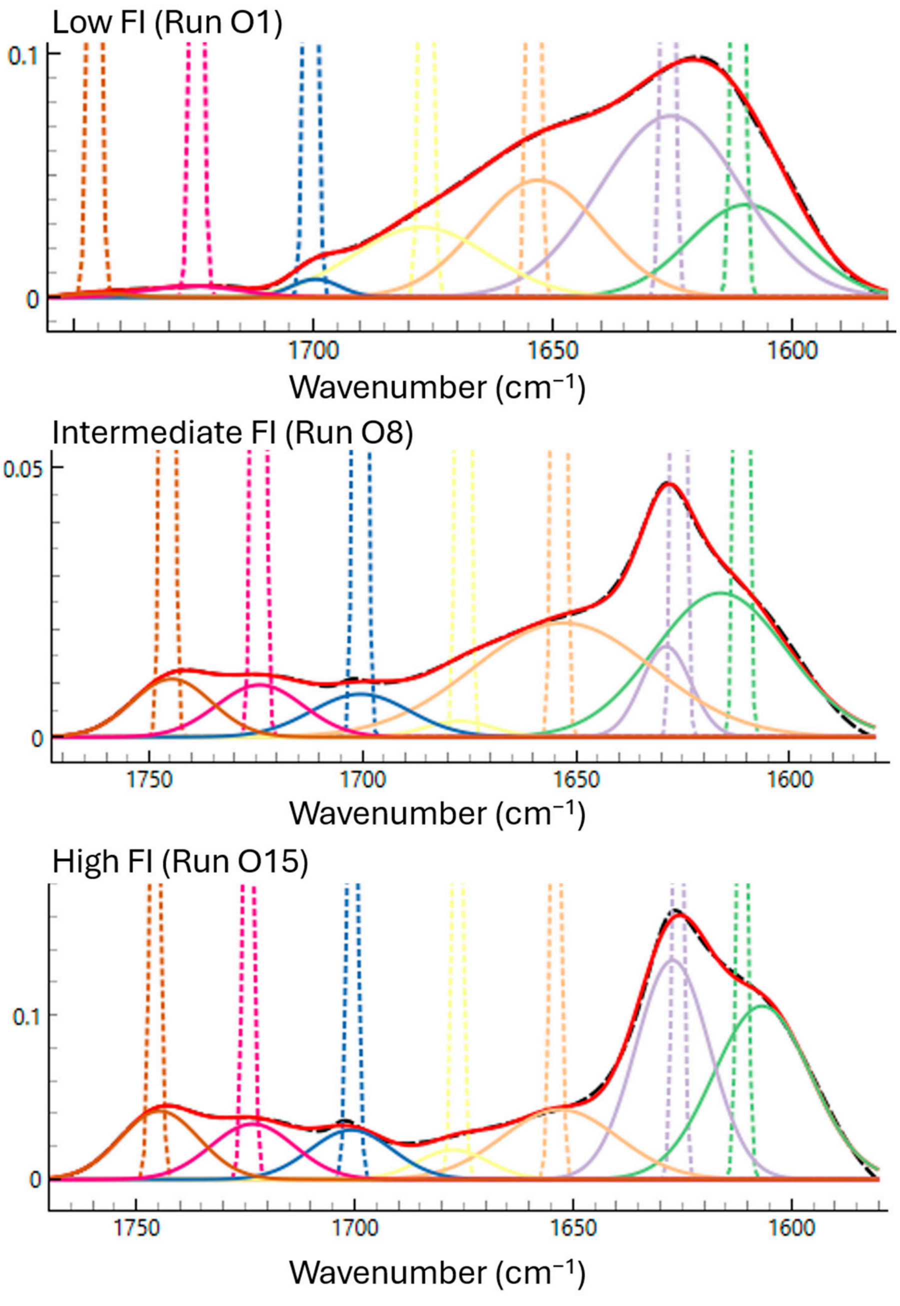

Figure S4. Representative constrained Gaussian deconvolutions of the FTIR Amide I region for samples exhibiting low (Run O1), intermediate (Run O8), and high (Run O15) Functionalization Index (FI) values. The experimental spectra (black) are shown together with the cumulative fitted spectra (red) and the individual Gaussian components. Peak centers were initialized from the second-derivative spectra and

fitted using the same constrained fitting strategy described in the Methods. The close agreement between the experimental and fitted spectra illustrates the robustness of the deconvolution procedure across the investigated range of functionalization.

S3.3. Screening Phase – Full Factorial Design (FFD)

Table S1. FTIR deconvolution parameters and calculated response variables obtained for the screening experiments (S1–S8). Peak positions were initialized from the minima of the second-derivative spectra and refined by constrained nonlinear Gaussian fitting, with peak centers allowed to vary within predefined peak-specific intervals (typically ±1 to ±5 cm^{-1}) around their initial positions. For each Gaussian component, the fitted center (cm^{-1}), area, standard deviation (σ, related to the full width at half maximum), and intensity are reported. Reduced χ^2 values are provided as goodness-of-fit metrics for the Gaussian deconvolution. FI and BI were calculated using both peak area- and intensity-based descriptors.

Parameter	S1	S2	S3	S4	S5	S6	S7	S8
**Center (cm^{-1})**	1610.61	1607.95	1609.54	1607.5	1606.23	1607.74	1606.45	1611.94
**Area**	0.671705	2.11961	1.13805	1.74442	0.877188	1.95602	4.79962	1.46541
**σ (cm^{-1})**	11.9206	10.0932	11.9814	9.57386	9.91225	10.284	10.2343	11.4403
**Intensity**	0.022479955	0.083780326	0.037894	0.072691	0.035305	0.07588	0.187096	0.051102
**Center (cm^{-1})**	1626.9	1626.01	1625.34	1626.58	1625.59	1625.82	1625.58	1627.78
**Area**	1.47084	9.67847	2.76126	7.95223	3.93652	5.40013	7.93289	2.21827
**σ (cm^{-1})**	11.0012	10.3914	14.7757	9.99897	11.0632	10.1226	9.42434	9.09099
**Intensity**	0.053338443	0.371575962	0.074555	0.317284	0.141954	0.212827	0.335811	0.097346
**Center (cm^{-1})**	1653.25	1654.41	1652.96	1652.96	1652.96	1652.96	1652.96	1652.96
**Area**	1.58305	5.86892	1.54902	5.25654	2.43941	4.13438	3.16763	2.67971
**σ (cm^{-1})**	14.3313	13.498	12.8093	13.0486	12.7815	14.607	13.299	15.8908
**Intensity**	0.044068068	0.173461753	0.048244	0.160713	0.076141	0.112918	0.095023	0.067276
**Center (cm^{-1})**	1679.96	1678.83	1677.39	1677.39	1677.39	1677.08	1677.39	1677.39
**Area**	0.542832	1.57398	1.02271	1.78386	0.977201	0.899035	0.74506	0.413981
**σ (cm^{-1})**	10.9438	8.87016	14.1283	9.67731	10.3467	8.81026	7.85396	8.48528
**Intensity**	0.019788472	0.070791762	0.028879	0.07354	0.037679	0.04071	0.037846	0.019464
**Center (cm^{-1})**	1699.59	1700.34	1699.47	1699.47	1699.58	1699.47	1700.34	1699.47
**Area**	0.23856	1.64985	0.0959816	1.35266	0.725822	2.0403	1.38462	1.15684
**σ (cm^{-1})**	7.77043	9.35985	5.09459	9.63999	9.20368	11.4939	9.85956	11.898
**Intensity**	0.012248067	0.07032189	0.007516	0.055979	0.031462	0.070818	0.056026	0.03879
**Center (cm^{-1})**	1745.3	1744.1	1744.64	1744.64	1744.64	1744.64	1744.64	1744.64
**Area**	0.409316	2.10681	0.0265709	1.55572	0.874665	1.74201	1.86956	1.17549
**σ (cm^{-1})**	9.0918	9.3179	6.01981	9.20322	9.28176	10.1575	9.36761	10.1387
**Intensity**	0.017960723	0.090203272	0.001761	0.067438	0.037595	0.068419	0.079621	0.046254
**Reduced χ^2**	1.23E-07	6.54E-06	5.32E-06	3.16E-06	5.79E-07	1.71E-06	7.83E-06	5.91E-07

**FI Area**	0.090818	0.100849	0.004046	0.086	0.097661	0.120723	0.103693	0.148155
**BI Area**	0.379278	0.542263	0.435089	0.514375	0.520575	0.515627	0.516783	0.425387
**FI Intensity**	0.118223	0.117157	0.008935	0.099144	0.116558	0.133331	0.111858	0.168825
**BI Intensity**	0.431709	0.573944	0.416417	0.54875	0.537656	0.552749	0.550486	0.496887

S3.4. Optimization Phase – Box-Behnken Design (BBD)

Table S2. FTIR deconvolution parameters and calculated response variables obtained for the optimization experiments (O1–O15). Peak positions were initialized from the minima of the second-derivative spectra and refined by constrained nonlinear Gaussian fitting, with peak centers allowed to vary within predefined peak-specific intervals (typically ±1 to ±5 cm^{-1}) around their initial positions. For each Gaussian component, the fitted center (cm^{-1}), area, standard deviation (σ, related to the full width at half maximum), and intensity are reported. Reduced χ^2 values are provided as goodness-of-fit metrics for the Gaussian deconvolution. FI and BI were calculated using both peak area- and intensity-based descriptors.

Parameter	O1	O2	O3	O4	O5	O6	O7	O8	O9	O10	O11	O12	O13	O14	O15
**Center (cm^{-1})**	1609.54	1607.5	1610.87	1611.33	1609.37	1608.5	1607.02	1616.14	1607.89	1616.23	1607.56	1616.23	1611.69	1616.23	1606.69
**Area**	1.13805	1.74442	3.03053	2.96581	1.51781	0.393707	1.80398	1.0253	1.00516	0.887305	0.956556	0.773806	0.30777	0.706944	2.90975
**σ (cm^{-1})**	11.9814	9.57386	11.0946	10.4797	10.9058	9.64013	8.85188	15.4531	11.6348	15.9246	11.3102	14.9356	10.6425	14.5717	11.096
**Intensity**	0.037893851	0.072690716	0.108973752	0.112904029	0.055523246	0.016293156	0.08130386	0.026469778	0.034466028	0.022228971	0.033740777	0.020669234	0.011537125	0.019354848	0.104617465
**Center (cm^{-1})**	1625.34	1626.58	1622.91	1627.97	1628.91	1628.91	1625.98	1628.91	1628.41	1628.91	1628.91	1628.91	1628.91	1628.91	1627.16
**Area**	2.76126	7.95223	16.1193	6.46469	4.2378	1.49145	9.4478	0.244142	0.729628	0.281834	0.773469	0.272622	0.507646	0.286323	2.8262
**σ (cm^{-1})**	14.7757	9.99897	12.1735	9.13959	9.49811	11.1266	9.7773	5.77754	7.92463	6.20686	8.11315	6.36333	8.45837	6.70357	8.45088
**Intensity**	0.074554558	0.317284336	0.528257513	0.282186319	0.177999272	0.05347628	0.38550207	0.016858328	0.036731398	0.018114919	0.038033682	0.017091942	0.023943589	0.017039822	0.13341846
**Center (cm^{-1})**	1652.96	1652.96	1652.96	1652.96	1652.96	1652.96	1652.96	1652.96	1652.96	1652.96	1652.96	1652.96	1652.96	1652.96	1652.96
**Area**	1.54902	5.25654	7.9688	7.87894	4.57797	1.23193	6.8133	1.11634	1.50736	1.11627	1.35874	1.06901	0.689474	1.01232	1.41176
**σ (cm^{-1})**	12.8093	13.0486	11.1168	14.5572	12.5078	12.4903	13.3981	21.0269	19.7681	21.4405	19.0601	21.3582	15.1185	20.9281	13.2414
**Intensity**	0.048244365	0.160713004	0.28597502	0.215925985	0.146018196	0.039348495	0.202875341	0.0211805	0.030420547	0.020770613	0.028439775	0.019967886	0.018193831	0.019297586	0.042534559
**Center (cm^{-1})**	1677.39	1677.39	1675.39	1677.39	1677.39	1677.39	1677.39	1677.39	1677.39	1677.39	1677.39	1677.39	1677.39	1677.39	1677.39
**Area**	1.02271	1.78386	3.54707	1.72268	1.08355	0.815126	1.48044	0.0584827	0.0927031	0.0355387	0.0907735	0.0333655	0.124785	0.0308355	0.363884
**σ (cm^{-1})**	14.1283	9.67731	10.5979	9.4506	9.33307	12.579	8.69224	7.8609	7.67183	6.59307	7.71175	6.61259	9.18449	6.41686	8.0463
**Intensity**	0.028878695	0.073539569	0.133525713	0.072721059	0.046316891	0.025851967	0.067947591	0.002968042	0.004820702	0.002150447	0.004695924	0.002012987	0.005420287	0.001917094	0.018041876
**Center (cm^{-1})**	1699.47	1699.47	1699.47	1699.47	1699.47	1699.47	1699.47	1700.39	1700.59	1701.46	1701.47	1700.3	1699.47	1699.47	1700.74
**Area**	0.0959816	1.35266	1.72189	2.0671	1.0468	0.352866	2.59921	0.229207	0.468621	0.257377	0.42348	0.219168	0.215482	0.205028	0.705781
**σ (cm^{-1})**	5.09459	9.63999	9.18941	11.8672	11.0449	9.28844	11.1891	11.474	12.8704	12.4091	13.2395	12.0821	12.6547	12.0288	9.36559
**Intensity**	0.00751612	0.055979244	0.074753708	0.069490941	0.037810889	0.015155909	0.092674698	0.007969443	0.014525954	0.008274551	0.012760752	0.007236852	0.006793195	0.006799952	0.030064209
**Center (cm^{-1})**	1744.64	1744.64	1744.64	1744.64	1744.64	1744.64	1744.64	1744.64	1745.08	1744.64	1744.66	1744.64	1744.64	1744.64	1744.64
**Area**	0.0265709	1.55572	2.29598	1.55177	0.903459	0.684195	2.09798	0.251338	0.299545	0.295767	0.346903	0.258945	0.191851	0.253775	0.936166
**σ (cm^{-1})**	6.01981	9.20322	9.20403	9.06642	9.0643	10.5015	9.77788	9.2913	9.01835	9.64101	8.9991	9.66811	9.59388	9.67299	8.96883
**Intensity**	**0.001760915**	**0.067438297**	**0.099518779**	**0.068282039**	**0.039763916**	**0.025992229**	**0.085599574**	**0.010791868**	0.01325104	0.012238893	0.015378853	0.010685157	0.007977829	0.010466538	0.041642044
**Reduced χ^2**	5,32E-06	3,16E-06	3,31E-05	4,37E-06	2,52E-06	5,90E-08	6,69E-06	2,27E-07	2,55E-07	4,50E-07	2,84E-07	1,95E-07	5,23E-08	1,54E-07	2,80E-06
**FI Area**	0.004046111	0.086000273	0.070890733	0.073546321	0.072485885	0.159669168	0.094739471	0.094011842	0.078755672	0.11471286	0.096281215	0.10935309	0.103975434	0.113219096	0.113925189
**BI Area**	0.435089417	0.514374747	0.550865007	0.404365185	0.42399147	0.4304042	0.544012503	0.177054053	0.315040828	0.20913231	0.332207287	0.207684087	0.391905946	0.219211176	0.429818646
**FI Intensity**	0.008934683	0.099143805	0.087954075	0.09065251	0.085759365	0.173136315	0.103094312	0.143040786	0.109544746	0.171078818	0.130693753	0.159530194	0.121081625	0.162500416	0.126696113
**BI Intensity**	0.416417284	0.548750089	0.532937551	0.466893297	0.465440642	0.457164498	0.575905959	0.329079623	0.423738349	0.368879701	0.431665177	0.363230715	0.466500301	0.370129366	0.497396785

Table S3. Reproducibility assessment of the FTIR deconvolution workflow based on five independent Gaussian fits of a representative spectrum. Individual fitting parameters, reduced χ^2 values, and the resulting FI and BI descriptors are reported. Mean, standard deviation (SD), and relative standard deviation (RSD) are provided for the calculated descriptors and reduced χ^2 values.

Parameter	O2 - 1	O2 - 2	O2 - 3	O2 - 4	O2 - 5
**Center (cm⁻¹)**	1607.5	1608.97	1609.15	1609.34	1609.27
**Area**	1.74442	2.10744	2.15207	2.20328	2.18404
**σ (cm⁻¹)**	9.57386	10.0048	10.0608	10.074	10.1006
**Intensity**	0.072690716	0.084035	0.085337	0.087253	0.086264
**Center (cm⁻¹)**	1626.58	1626.54	1626.57	1626.51	1626.59
**Area**	7.95223	6.91749	6.83108	6.5129	6.77289
**σ (cm⁻¹)**	9.99897	9.52188	9.47947	9.33488	9.4506
**Intensity**	0.317284336	0.289828	0.287488	0.278343	0.28591
**Center (cm⁻¹)**	1652.96	1652.46	1652.36	1651.81	1652.29
**Area**	5.25654	6.42077	6.46969	6.81127	6.49796
**σ (cm⁻¹)**	13.0486	15.4564	15.5529	16.2847	15.6067
**Intensity**	0.160713004	0.165727	0.165954	0.166864	0.166104
**Center (cm⁻¹)**	1677.39	1679.95	1680.04	1680.97	1680.08
**Area**	1.78386	1.4954	1.50284	1.54639	1.50803
**σ (cm⁻¹)**	9.67731	9.76594	9.83362	10.389	9.87395
**Intensity**	0.073539569	0.061088	0.06097	0.059383	0.06093
**Center (cm⁻¹)**	1699.47	1699.72	1699.78	1700.26	1699.81
**Area**	1.35266	0.947781	0.925946	0.741839	0.915758
**σ (cm⁻¹)**	9.63999	8.05591	7.97474	7.2801	7.93838
**Intensity**	0.055979244	0.046936	0.046322	0.040653	0.046022
**Center (cm⁻¹)**	1744.64	1745.93	1745.94	1746.07	1745.94
**Area**	1.55572	1.13802	1.12934	1.02992	1.12625
**σ (cm⁻¹)**	9.20322	8.73032	8.72316	8.60889	8.72104
**Intensity**	0.067438297	0.052004	0.051649	0.047728	0.051521

Parameter	O2 - 1	O2 - 2	O2 - 3	O2 - 4	O2 - 5	Mean	SD	RSD (%)
**Reduced χ^2**	3.1563E-06	3.0181E-06	2.9658E-06	2.7149E-06	2.9299E-06	2.9570E-06	1.6037E-07	5.42
**FI Area**	0.0860003	0.0636161	0.0631564	0.0578098	0.0629940	0.06672	0.01104	16.54
**BI Area**	0.5143747	0.4396737	0.4337987	0.4072109	0.4300457	8.70160	0.04068	9.14
**FI Intensity**	0.0991438	0.0803004	0.0799440	0.0754594	0.0798485	0.08294	0.00927	11.18
**BI Intensity**	0.5487501	0.5200070	0.5166772	0.5043441	0.5144394	0.52084	0.01666	3.20

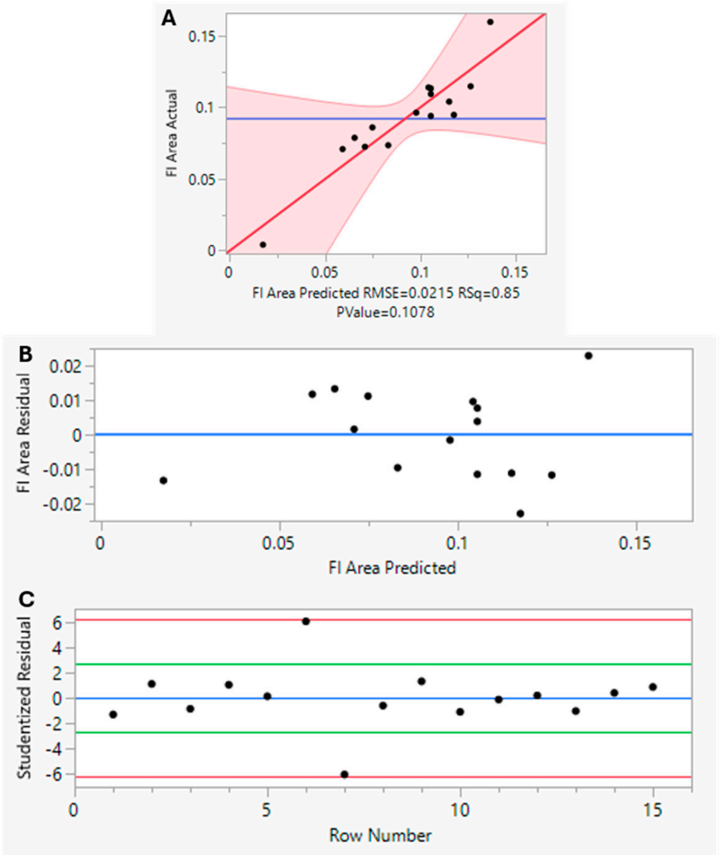

D

Source	DF	Sum of Squares	Mean Square	F Ratio
Lack Of Fit	3	0.00211541	0.000705	6.8325
Pure Error	2	0.00020641	0.000103	**Prob > F**
Total Error	5	0.00232181		0.1303

E

Source	DF	Sum of Squares	Mean Square	F Ratio
Model	9	0.01329070	0.001477	3.1802
Error	5	0.00232181	0.000464	**Prob > F**
C. Total	14	0.01561251		0.1078

Figure S5. Diagnostic evaluation of the area-based FI response model. (A) Observed versus predicted values with 95% confidence bands; (B) residuals versus predicted values; (C) studentized residuals versus run order; (D) lack-of-fit analysis; and (E) analysis of variance (ANOVA). The model showed good agreement between observed and predicted responses (R^2 = 0.85, RMSE = 0.0215), while the residuals were randomly distributed around zero with no evident systematic trend, supporting the adequacy of the fitted model. The lack-of-fit test was not statistically significant (p = 0.1303), indicating that the quadratic model adequately described the experimental data within the investigated design space. Although the overall model was not statistically significant (ANOVA, p = 0.1078), this result is consistent with the exploratory nature of the FI response discussed in the manuscript.

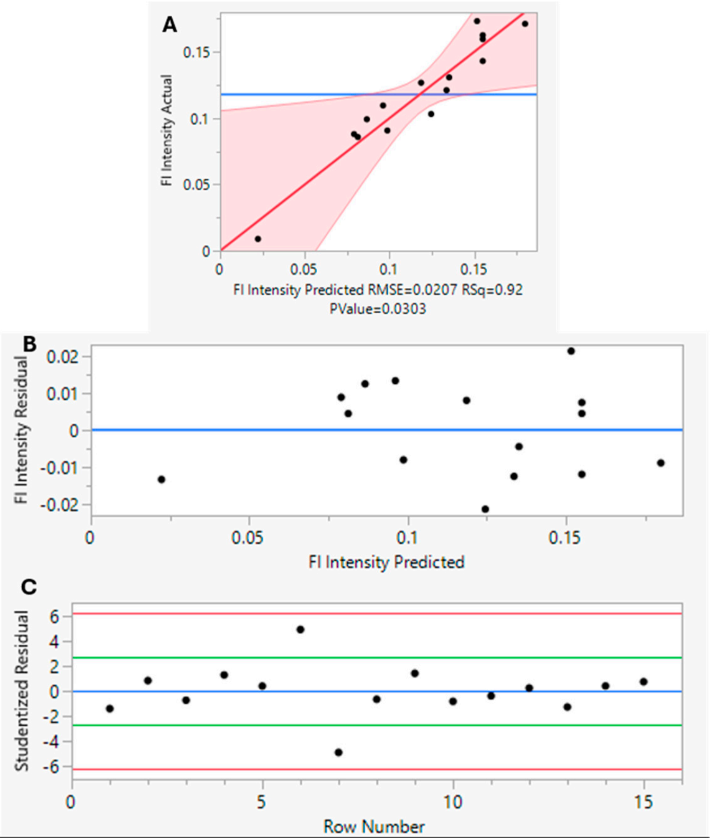

D

Source	DF	Sum of Squares	Mean Square	F Ratio
Lack Of Fit	3	0.00192393	0.000641	5.8354
Pure Error	2	0.00021980	0.000110	**Prob > F**
Total Error	5	0.00214373		0.1498

E

Source	DF	Sum of Squares	Mean Square	F Ratio
Model	9	0.02352601	0.002614	6.0969
Error	5	0.00214373	0.000429	**Prob > F**
C. Total	14	0.02566974		0.0303*

Figure S6. Diagnostic evaluation of the intensity-based FI response model. (A) Observed versus predicted values with 95% confidence bands; (B) residuals versus predicted values; (C) studentized residuals versus run order; (D) lack-of-fit analysis; and (E) analysis of variance (ANOVA). The model showed good agreement between observed and predicted responses (R^2 = 0.92, RMSE = 0.0207), while the residuals were randomly distributed around zero with no evident systematic trend, supporting the adequacy of the fitted model. The lack-of-fit test was not statistically significant (p = 0.1498), indicating that the quadratic model adequately described the experimental data within the investigated design space. The overall model was statistically significant (ANOVA, p = 0.0303), supporting the reliability of the fitted regression model.

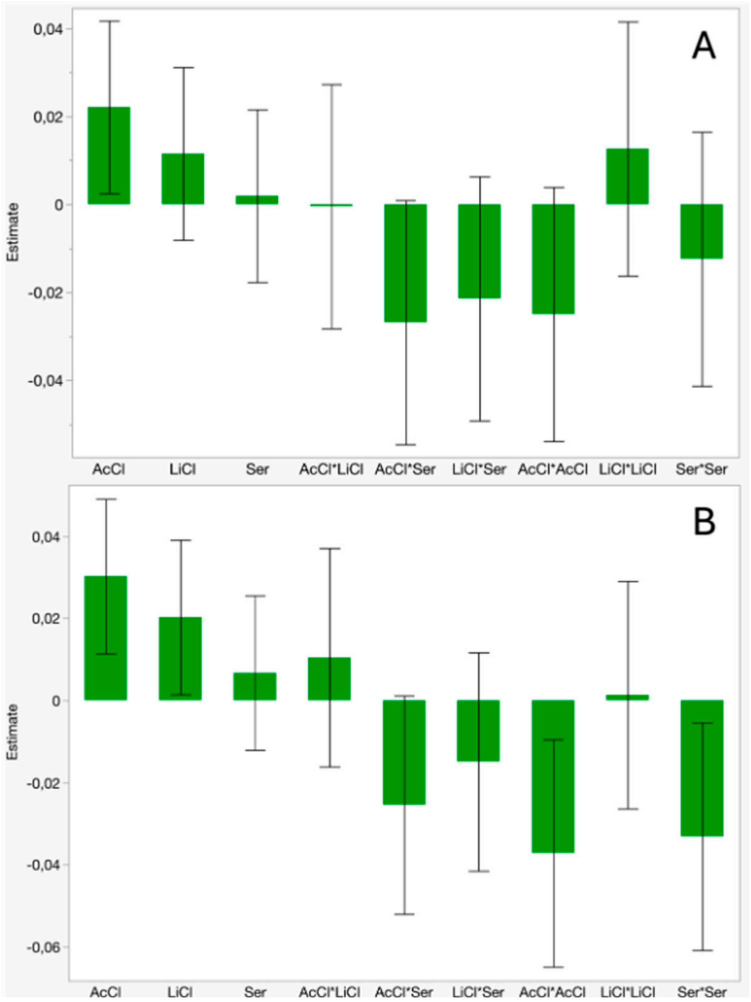

Figure S7. Coefficient plots illustrating the estimated effects of model terms on the FI obtained from the Box–Behnken design. (A) FI calculated from peak area and (B) FI calculated from peak intensity (amplitude). Error bars represent confidence intervals, and positive and negative coefficients indicate enhancing or reducing contributions to the FI response, respectively.

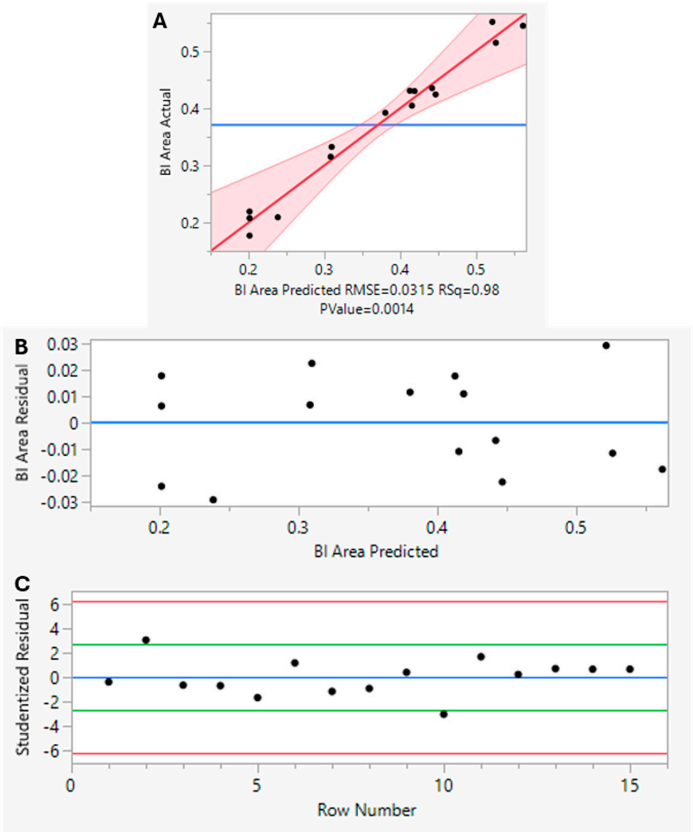

D

Source	DF	Sum of Squares	Mean Square	F Ratio
Lack Of Fit	3	0.00399931	0.001333	2.8082
Pure Error	2	0.00094943	0.000475	**Prob > F**
Total Error	5	0.00494875		0.2735

E

Source	DF	Sum of Squares	Mean Square	F Ratio
Model	9	0.21148399	0.023498	23.7416
Error	5	0.00494875	0.000990	**Prob > F**
C. Total	14	0.21643273		0.0014*

Figure S8. Diagnostic evaluation of the area-based BI response model. (A) Observed versus predicted values with 95% confidence bands; (B) residuals versus predicted values; (C) studentized residuals versus run order; (D) lack-of-fit analysis; and (E) analysis of variance (ANOVA). The model showed excellent agreement between observed and predicted responses (R^2 = 0.98, RMSE = 0.0014), while the residuals were randomly distributed around zero with no evident systematic trend, supporting the adequacy of the fitted model. The lack-of-fit test was not statistically significant (p = 0.2735), indicating that the quadratic model adequately described the experimental data within the investigated design space. The overall model was highly statistically significant (ANOVA, p = 0.0014), confirming the robustness and predictive capability of the fitted regression model.

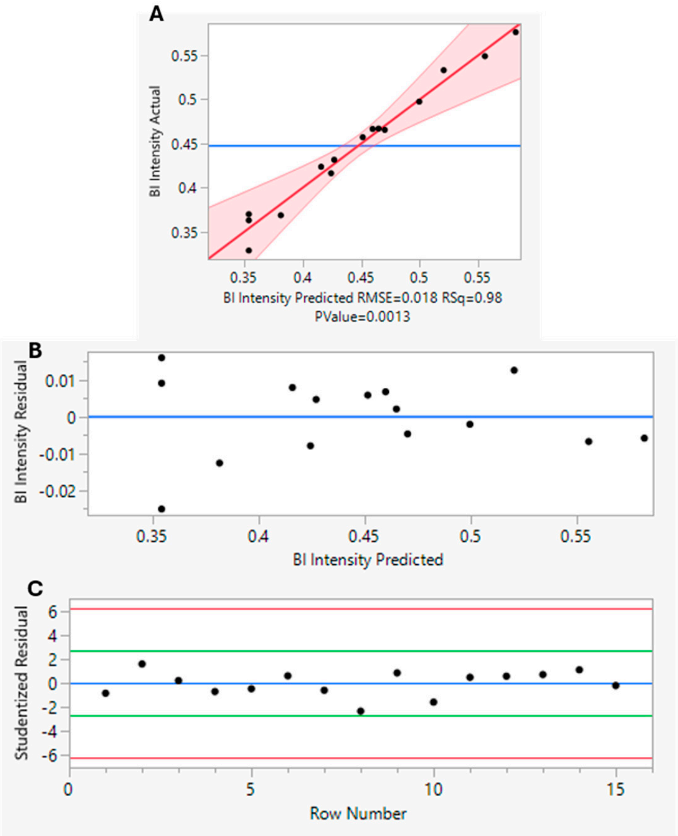

D

Source	DF	Sum of Squares	Mean Square	F Ratio
Lack Of Fit	3	0.00065324	0.000218	0.4507
Pure Error	2	0.00096632	0.000483	**Prob > F**
Total Error	5	0.00161956		0.7438

E

Source	DF	Sum of Squares	Mean Square	F Ratio
Model	9	0.07113513	0.007904	24.4014
Error	5	0.00161956	0.000324	**Prob > F**
C. Total	14	0.07275470		0.0013*

Figure S9. Diagnostic evaluation of the intensity-based BI response model. (A) Observed versus predicted values with 95% confidence bands; (B) residuals versus predicted values; (C) studentized residuals versus run order; (D) lack-of-fit analysis; and (E) analysis of variance (ANOVA). The model showed excellent agreement between observed and predicted responses ($R^2 = 0.98$, RMSE = 0.0018), while the residuals were randomly distributed around zero with no evident systematic trend, supporting the adequacy of the fitted model. The lack-of-fit test was not statistically significant ($p = 0.7438$), indicating that the quadratic model adequately described the experimental data within the investigated design space. The overall model was highly statistically significant (ANOVA, $p = 0.0013$), confirming the robustness and predictive capability of the fitted regression model.

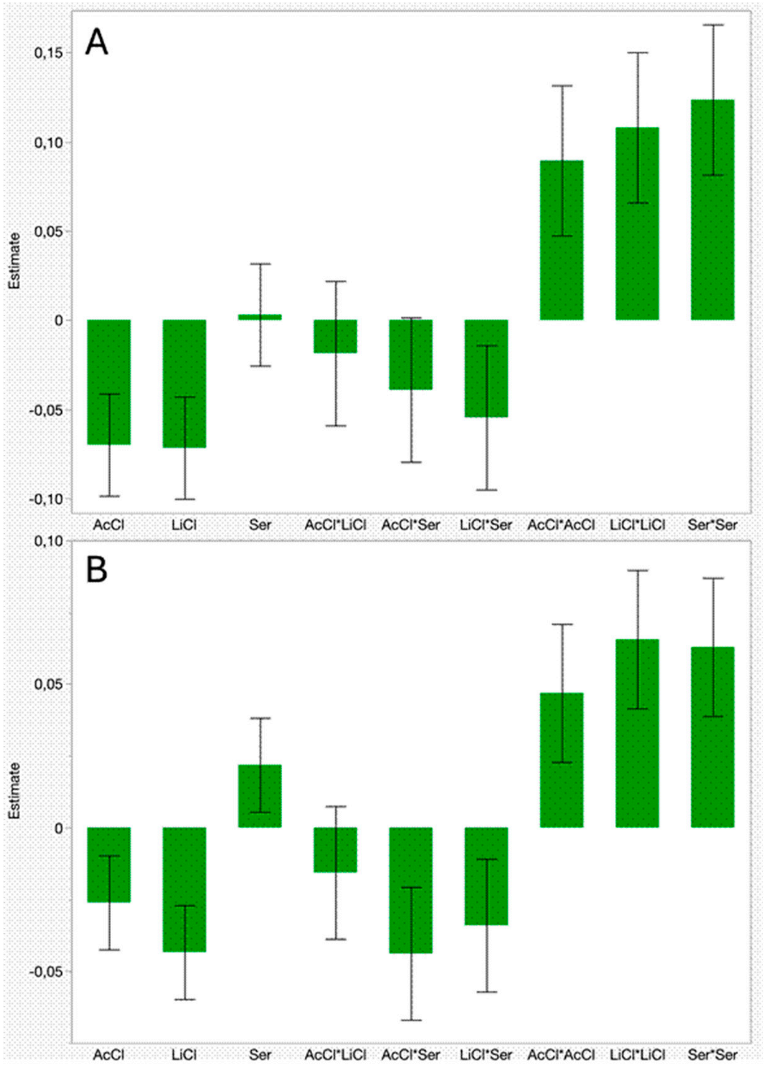

Figure S10. Coefficient plots illustrating the estimated effects of model terms on the BI obtained from the Box–Behnken design. (A) Results based on BI calculated from peak area and (B) results based on BI calculated from peak intensity (amplitude). Error bars represent confidence intervals, and positive and negative coefficients indicate enhancing or reducing contributions to the BI response, respectively.
